# Depression and Long‐Term HbA_1c_ Variability Are Additively Associated With Increased Risk of Cardiovascular–Renal Outcomes, Severe Hypoglycaemia and Mortality in Type‐2 Diabetes

**DOI:** 10.1002/dmrr.70222

**Published:** 2026-08-31

**Authors:** Kelly T. C. Wong, Edith W. K. Chow, Eric S. H. Lau, Chun Kwan O, Alice P. S. Kong, Ronald C. W. Ma, Andrea O. Y. Luk, Elaine Y. K. Chow, Yun Kwok Wing, Juliana C. N. Chan, Juliana N. M. Lui

**Affiliations:** ^1^ Department of Medicine and Therapeutics, Faculty of Medicine The Chinese University of Hong Kong Hong Kong SAR China; ^2^ Phase 1 Clinical Trial Centre The Chinese University of Hong Kong Hong Kong SAR China; ^3^ Hong Kong Institute of Diabetes and Obesity, The Chinese University of Hong Kong Hong Kong SAR China; ^4^ Li Ka Shing Institute of Health Science, The Chinese University of Hong Kong Hong Kong SAR China; ^5^ Department of Psychiatry, Faculty of Medicine The Chinese University of Hong Kong Hong Kong SAR China; ^6^ Asia Diabetes Foundation Hong Kong SAR China

**Keywords:** cancer, cardiovascular disease, chronic kidney disease, depression, HbA_1c_ variability, mortality, severe hypoglycaemia, type‐2 diabetes

## Abstract

**Aims:**

Depression and long‐term HbA_1c_ variability are associated with adverse outcomes in type‐2 diabetes (T2D), which may be related to suboptimal self‐management practices and health‐related quality of life (HRQoL).

**Materials and Methods:**

In the prospective Hong Kong Diabetes Register (2013–2019), we estimated the independent and joint associations of depression and HbA_1c_ variability with outcomes. Depression was measured using the Patient Health Questionnaire‐9 (PHQ‐9) with a validated Chinese cut‐off score ≥ 7. The HbA_1c_ variability score (HVS) was expressed as the percentage of HbA_1c_, which varied by 0.5% compared to the preceding value.

**Results:**

Amongst 5657 Chinese patients with T2D [58.7% male, age (mean ± SD): 58.47 ± 10.62 years, duration of diabetes: 10.63 ± 8.19 years and time‐weighted HbA_1c_: 7.44% ± 1.04%], 13.3% had depression. After full adjustment, depression was associated with increased risk of cardiovascular disease (CVD) [adjusted hazard ratio (aHR): 1.79 (95% CI: 1.15–2.79); *p* = 0.010], whereas high HVS (≥ median, 42.9%) was associated with increased risks of chronic kidney disease (CKD) [1.49 (1.21–1.82); *p* < 0.001] and severe hypoglycaemia (SH) [1.93 (1.22–3.07); *p* = 0.005]. The depression‐high‐HVS group had the highest aHR for CKD, which was attenuated from 1.90 (1.41–2.56) to 1.74 (1.25–2.44), and that for all‐cause mortality from 2.00 (1.33–3.01) to 1.72 (1.06–2.78) after adjustment for self‐management practices and HRQoL.

**Conclusions:**

Depression and high HVS are independently and differentially associated with adverse events, with the depression‐high‐HVS group having the worst outcomes, which were modulated by self‐management and HRQoL, calling for systematic screening of depression and holistic management aimed at avoiding fluctuating glycaemic control in T2D.

## Graphical abstract

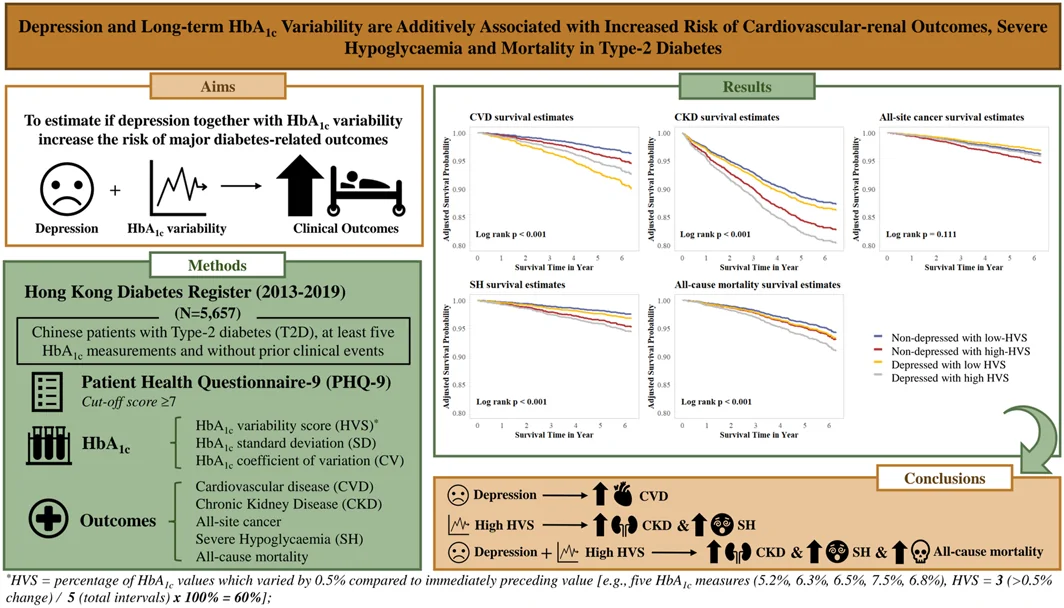

## 1 Introduction

Depression is defined as a prolonged period of persistent sadness with a marked reduction in interest or pleasure in previously enjoyable activities [1]. Depression and type‐2 diabetes (T2D) are chronic diseases that frequently coexist with increased disease burden [2]. Our group previously reported that patients with T2D comorbid depression had 2‐fold higher risk of cardiovascular disease (CVD) and 54% increased risk of all‐cause mortality [3]. Both diabetes and depression have complex aetiologies, including genetic, perinatal, socio‐psychological, behavioural and neurohormonal factors, which interact to cause a myriad of phenotypes, trajectories and outcomes [4]. Biologically, depression is associated with metabolic abnormalities, activation of immunoinflammatory pathways and increased activity of counter‐regulatory hormones [5]. Self‐management practices include the adoption of a healthy lifestyle, regular medical consultations and adherence to medications [6]. Patients with comorbid diabetes and depression might face challenges in adopting these behavioural changes, leading to suboptimal glycaemic control and high rates of hospitalisation and complications [7, 8]. In this light, the American Diabetes Association recommended evaluation of patient‐reported outcomes (PROs) to capture patients' perception of their psychological status and provision of holistic care to improve physical and mental health in patients with diabetes [6].

Loss of beta‐cell function and insulin resistance can lead to fluctuating glycaemic control or glycaemic variability (GV) with frequent episodes of hyper and hypoglycaemia, which can be worsened by behavioural factors. Mechanistically, high GV can cause abnormal internal milieu with increased inflammation, oxidative stress, endothelial dysfunction and abnormal cell cycle [9, 10]. There are also bidirectional associations between depression and GV in individuals with or without diabetes [11, 12, 13]. Self‐monitored blood glucose (SMBG) and continuous glucose monitoring (CGM) systems provide information on short‐term GV [14, 15]. Visit‐to‐visit measurements of glycated haemoglobin (HbA_1c_) are a common measure of long‐term GV [16, 17]. These can be indicated by HbA_1c_ variability score (HVS), expressed as the percentage of HbA_1c_ values that varied by 0.5% (higher or lower) than the preceding values. Other indicators include standard deviation (SD) and coefficient and variation (CV) of HbA_1c_.

Using the prospective Hong Kong Diabetes Register (HKDR), which captured all laboratory results of enroled patients, we have reported that one SD of HbA_1c_ was associated with 16% and 27% increased risk of chronic kidney disease (CKD) and CVD, respectively [18]. In another study, patients with T2D, obesity and high HVS had 42% increased risk of all‐site cancers compared to their non‐obese counterparts with low‐GV [19]. Given the importance of self‐management and health‐related quality of life (HRQoL) on clinical outcomes [6], we hypothesise that depression might impede self‐management and HRQoL with high GV leading to increased incidence of CVD, CKD, all‐site cancer, severe hypoglycaemia (SH) and all‐cause mortality in patients with T2D.

## 2 Materials and Methods

### 2.1 Study Population and Setting

**FIGURE 1:**
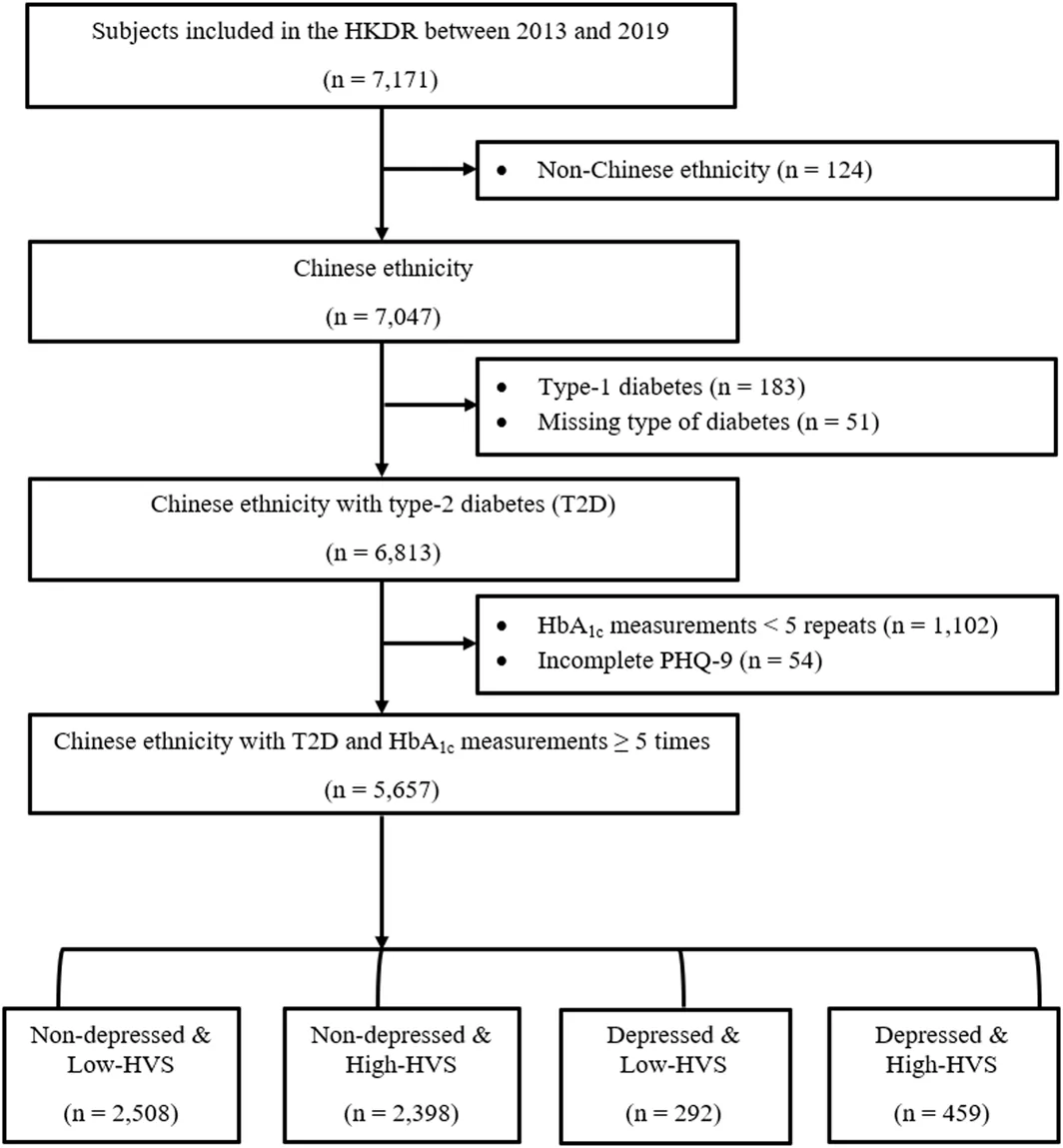
CONSORT diagram of patient selection for analysis, with a stratification of four patient groups by depressed versus non‐depressed and high versus low HbA_1c_ variability score (HVS), in the Hong Kong Diabetes Register (HKDR). PHQ‐9, patient health questionnaire‐9.

The Hong Kong Diabetes Register (HKDR), established since 1995 at the Prince of Wales Hospital (PWH), is an ongoing research‐driven quality improvement programme where 22,825 patients with diabetes underwent periodic comprehensive assessment of risk factors and complications at the Diabetes Centre. Recognising the importance of holistic assessment on psychological well‐being, we incorporated assessment on health‐related quality of life (HRQoL)—EuroQol 5‐Dimension 3‐Level (EQ‐5D‐3L) in 2007 and depression—Patient Health Questionnaire‐9/(PHQ‐9) in 2013. A total of 7171 patients enroled in the HKDR between 2013 and 2019 with complete EQ‐5D‐3L and PHQ‐9 survey results were included in this study. Exclusion criteria included type‐1 diabetes [acute presentation with heavy ketonuria (> 3+) or ketoacidosis, continuous insulin treatment within one year of diagnosis], unknown diabetes type, non‐Chinese ethnicity and fewer than five HbA_1c_ measurements. For outcome analyses, patients with prior events (CVD, CKD, all‐site cancer and SH) were excluded. Figure 1 shows the CONSORT diagram of patient selection.

## 3 Baseline Measurements

### 3.1 Patient‐Reported Outcome Measures

Depressive symptoms were measured by PHQ‐9 [20] with validation in Chinese patients with T2D based on interviews with psychiatrists [21]. It measures the presence and severity of depressive symptoms in the past 2 weeks, and consists of nine items with reference to the Diagnostic and Statistical Manual of Mental Disorders (DSM‐IV). The score ranges from 0 (*not at all*) to 3 (*nearly every day*), with a total score of 27. Validated in Chinese patients with T2D, a score of 7 identified significant depression with a sensitivity of 82.6% and a specificity of 73.7% [21]. The good internal consistency (Cronbach's *α* of 0.83) in the present study. We measured HRQoL using EQ‐5D‐3L instrument [22]. It comprises five dimensions, namely mobility, self‐care, usual activities, pain/discomfort and anxiety/depression, with a self‐reported scale for each dimension ranging from 1 (*no problem*) to 3 (*extreme problem*). Since depression was measured by PHQ‐9, we excluded the dimension of anxiety/depression in EQ‐5D‐3L in this analysis.

### 3.2 Laboratory Results

During the protocol‐driven assessment, trained nurses used case report forms to document patients' demographics, socio‐economic factors, medications, medical history, self‐care practices and lifestyle habits followed by physical examination. Fasting blood samples were collected for measurement of HbA_1c,_ lipid profiles [total cholesterol, triglycerides, low‐density lipoprotein cholesterol (LDL‐C) and high‐density cholesterol (HDL‐C)] and renal/liver functions. Urinary albumin‐to‐creatinine ratio (uACR) was measured using random spot urine samples. Estimated glomerular filtration rate (eGFR) was calculated using the Chronic Kidney Disease Epidemiology Collaboration (CKD‐EPI) equation [23] (Supporting Information S1: Table S1). The Hospital Authority provides over 90% of inpatient services in Hong Kong, supported by a territory‐wide electronic medical record (EMR) system. We retrieved HbA_1c_ measurements from the enrolment date to the first occurrence date of CVD, CKD, all‐site cancer, SH, all‐cause mortality or the censor date of 31st December 2019, whichever came earlier from the HA‐EMR. The HbA_1c_ measurement 2 years prior to the clinical event were excluded from the analysis to avoid reverse causality.

### 3.3 Definition of Outcomes

CVD was identified by coronary heart disease (CHD) [ICD‐9 code 410, 412, angina pectoris (ICD‐9 code 413) or ischaemic heart disease (ICD‐9 code 411, 414)] and stroke [ICD‐9 code 430–434, or cerebrovascular disease (ICD‐9 code 436–437)]. CKD was defined by at least two values of eGFR < 60 mL/min per 1.73 m^2^, separated by 90–365 days (excluding in‐patient measurement during acute kidney injury). All‐site cancer was identified by neoplasms (ICD‐9 code 140–208). SH was defined as one or more hospitalisation for hypoglycaemia (ICD‐9 code 251.2) in the 12 months before/at enrolment or during the follow‐up period, including coma (ICD‐9 code 250.3) and other specified manifestations (ICD‐9 code 250.8).

### 3.4 Statistical Analysis

Data are expressed as mean ± standard deviation (SD), median [interquartile range (IQR)] or percentages. Three indexes are used to express GV: HVS, standard deviation (HbA_1c_‐SD) and coefficient of variation (HbA_1c_‐CV). HVS was calculated as the percentage of HbA_1c_ values that varied by 0.5% (larger or smaller) compared to the immediately preceding value; HbA_1c_‐SD, the square root of the variance and HbA_1c_‐CV, the ratio of SD to the sample mean. The median values of HVS, HbA_1c‐_SD and HbA_1c_‐CV were used to define high or low GV. Depression was defined by PHQ‐9 ≥ 7 [21]. Using HVS in the primary analysis, patients were stratified into 4 groups: (1) depressed‐high‐HVS (8%); (2) depressed‐low‐HVS (5%); (3) non‐depressed‐high‐HVS (42%); and (4) non‐depressed‐low‐HVS (45%) as the referent group. Time‐weighted mean HbA_1c_ was calculated using area under curve (AUC) with trapezoidal integration.

Multivariate cox regression models were employed to explore the independent and joint risk associations of HVS and depression on clinical outcomes adjusted for confounders. Based on prior knowledge, we divided confounders into 4 categories that were entered into the model in a stepwise approach. Model 1 adjusted for demographic and clinical factors followed by sequential adjustment on medication use (Model 2), self‐management behaviours (Model 3), HRQoL (Model 4) and depression or GV (HVS/HbA_1c_‐SD/HbA_1c_‐CV) (Model 5) (Supporting Information S1: Table S2). Results were expressed as adjusted hazard ratio (aHR) and 95% confidence interval (CI), with interaction terms included as appropriate. Statistical analyses were performed using R software (version 4.3.0). A *p*‐value less than 0.05 (2‐sided) was considered significant.

## 4 Results

Amongst the 7171 subjects enroled from HKDR between 2013 and 2019 invited to complete the PHQ‐9 questionnaire, 5657 Chinese patients with T2D [58.7% men, mean age of 58.5 ± 10.6 years, and median diabetes duration of 9.0 (4.0, 16.0) years] with complete data were included in the analysis. In this cohort, the median HbA_1c_ measurements per patient was 18.0 (10.0, 29.0) times with a time‐weighted HbA_1c_ of 7.4% ± 1.0%. Excluded patients due to limited HbA_1c_ measures and prior events were younger and had poorer lifestyle and self‐management practices, worse risk profiles and higher event rates than patients included in the analysis (Supporting Information S1: Table S3). After a median follow period of 5.7 (4.9, 6.4) years, the cumulative incidence expressed as per 1000 patient‐years was 6.9 for CVD, 23.4 for CKD, 6.2 for all‐site cancer, 5.3 for SH, and 9.2 for all‐cause mortality, respectively.

### 4.1 Baseline Characteristics and Event Rates by Depression and HbA_1c_ Variability Status

In this cohort, 13.3% had depression (PHQ‐9 ≥ 7) and 49.5% had HVS above the median value (42.9%). In the depressed group, 61.1% had high HVS. In the non‐depressed group, 48.9% had high HVS. In the high HVS group, 16.1% had depression compared with 10.4% in the low HVS group. Compared with their respective counterparts, participants with depression and high HVS were younger at diabetes diagnosis, had longer disease duration, and were less likely to have attained college education. Both groups were more physically inactive and exhibited poorer cardiometabolic risk profiles and lower HRQoL. They were more intensively treated with greater insulin use but reported lower medication adherence. Patients with depression also had a higher time‐weighted HbA_1c_ levels.

During the 5‐year follow up, both the depressed and high HVS groups had higher incident rates for all outcomes, including all‐cause mortality, compared with their counterparts (Supporting Information S1: Table S4). The incidence of all‐site cancers was similar between participants with and without depression.

Across the four stratified groups, the highest incidence of all‐site cancers was observed in the non‐depressed‐high‐HVS group, whereas the highest incidence of other outcomes (CVD, CKD, SH and all‐cause mortality) occurred in the depressed‐high‐HVS group (Table 1).

**TABLE 1:** Clinical characteristics of patients with type 2 diabetes stratified by PHQ9 ≥ 7 and median value of HVS (≥ 42.9%).

	Frequency (%)/Mean ± SD/Median (IQR)				*p*‐value^*^
	Non‐depressed and low‐HVS (*n* = 2508)	Non‐depressed and high‐HVS (*n* = 2398)	Depressed and low‐HVS (*n* = 292)	Depressed and high‐HVS (*n* = 459)	
Demographics, socio‐economic status and social habits					
Age (years)	59.3 ± 10.0	57.7 ± 11.2	58.5 ± 10.4	57.9 ± 10.9	**<** **0.001**
Men (*n*, %)	1495 (59.6)	1450 (60.5)	135 (46.2)	242 (52.7)	**<** **0.001**
College or above (*n*, %)	485 (19.4)	352 (14.8)	37 (12.8)	51 (11.2)	**<** **0.001**
Current smoker (*n*, %)	213 (8.5)	323 (13.5)	24 (8.2)	63 (13.7)	**<** **0.001**
Never alcohol drinker (*n*, %)	1473 (58.7)	1417 (59.1)	197 (67.5)	291 (63.4)	**0.013**
Adherence to balanced diet in last 3 months (*n*, %)	1309 (52.4)	1252 (52.4)	145 (49.7)	240 (52.6)	0.837
Physical activity 5 times or more per week in last 3 months (*n*, %)	945 (37.8)	816 (34.4)	81 (27.9)	127 (28.3)	**<** **0.001**
Metabolic risk factors					
Age at diagnosis (years)	51.0 (44.0, 58.0)	46.0 (38.0, 52.0)	50.5 (42.8, 57.0)	45.0 (37.0, 52.0)	**<** **0.001**
Duration of diabetes (years)	7.0 (3.0, 13.0)	12.0 (6.0, 18.0)	7.0 (3.0, 12.0)	13.0 (7.0, 18.5)	**<** **0.001**
Fasting plasma glucose (mmol/L)	7.0 ± 1.7	8.2 ± 2.78	7.0 ± 1.9	8.5 ± 3.8	**<** **0.001**
Time‐weighted mean HbA_1c_ (%)	6.9 ± 0.7	8.0 ± 1.0	6.9 ± 0.7	8.2 ± 1.2	**<** **0.001**
BMI (kg/m^2^)	26.0 ± 4.5	27.0 ± 4.9	26.6 ± 5.1	27.2 ± 4.8	**<** **0.001**
Waist‐hip‐ratio (women)	0.91 ± 0.06	0.93 ± 0.07	0.92 ± 0.08	0.95 ± 0.08	**<** **0.001**
Waist‐hip‐ratio (men)	0.96 ± 0.06	0.98 ± 0.07	0.98 ± 0.07	0.98 ± 0.07	**<** **0.001**
SBP (mmHg)	131.9 ± 17.3	134.1 ± 18.0	131.6 ± 17.9	137.1 ± 20.1	**<** **0.001**
DBP (mmHg)	75.0 ± 10.6	75.2 ± 11.1	75.3 ± 11.7	76.0 ± 11.2	0.356
HDL‐C (mmol/L)	1.4 ± 0.4	1.3 ± 0.4	1.3 ± 0.4	1.3 ± 0.4	**<** **0.001**
LDL‐C (mmol/L)	2.2 ± 0.7	2.3 ± 0.7	2.3 ± 0.8	2.2 ± 0.8	0.064
Triglyceride (mmol/L)	1.2 (0.9, 1.8)	1.3 (1.0, 1.9)	1.4 (1.0, 2.0)	1.4 (1.0, 2.0)	**<** **0.001**
uACR (mg/mmoL)	1.2 (0.5, 3.9)	2.6 (0.8, 12.4)	1.4 (0.6, 6.6)	4.1 (1.1, 32.2)	**<** **0.001**
eGFR (mL/min/1.73 m^2^)	85.6 ± 19.6	82.6 ± 25.2	85.2 ± 21.2	77.0 ± 30.6	**<** **0.001**
Medications (*n*, %)					
Oral glucose lowering drugs	2165 (86.3)	2193 (91.5)	254 (87.0)	400 (87.1)	**<** **0.001**
Insulin	409 (16.3)	1149 (47.9)	42 (14.4)	283 (61.7)	**<** **0.001**
Lipid lowering drugs	1680 (67.0)	1634 (68.1)	190 (65.1)	332 (72.3)	0.102
Blood pressure lowering drugs	1789 (71.3)	1838 (76.6)	214 (73.3)	367 (80.0)	**<** **0.001**
Statin	1580 (64.1)	1512 (63.9)	180 (62.9)	304 (67.3)	0.54
Renin angiotensin system inhibitor	1282 (51.7)	1486 (62.5)	153 (52.6)	285 (63.2)	**<** **0.001**
Incident of complications and comorbidities (*n*, %)					
CVD	123 (4.9)	195 (8.1)	22 (7.5)	66 (14.4)	**<** **0.001**
CKD	503 (20.1)	758 (31.6)	63 (21.6)	191 (41.6)	**<** **0.001**
All‐site cancer	90 (3.6)	125 (5.2)	7 (2.4)	14 (3.1)	**0.006**
SH	44 (1.8)	146 (6.1)	9 (3.1)	45 (9.8)	**<** **0.001**
All‐cause mortality	85 (3.4)	144 (6.0)	15 (5.1)	54 (11.8)	**<** **0.001**
Patient‐reported outcomes					
PHQ‐9 (0–27)	1.0 (0.0, 3.0)	1.0 (0.0, 3.0)	9.0 (7.0, 11.0)	9.0 (7.5, 12.0)	**<** **0.001**
EQ‐5D‐3L (dimension 1–4) (*n*, %)					
No problems in walking about	2378 (94.8)	2239 (93.4)	222 (76.0)	334 (72.8)	**<** **0.001**
No problems with self‐care	2474 (98.6)	2345 (97.8)	266 (91.1)	425 (92.6)	**<** **0.001**
No problems with performing usual activities	2410 (96.1)	2283 (95.2)	222 (76.0)	335 (73.0)	**<** **0.001**
No pain or discomfort	1619 (64.6)	1535 (64.0)	76 (26.0)	148 (32.2)	**<** **0.001**
Self‐reported medication adherence (0–100)	100.0 (90.0, 100.0)	100.0 (90.0, 100.0)	99.0 (90.0, 100.0)	95.0 (80.0, 100.0)	**<** **0.001**
Self‐manage blood glucose (*n*, %)	1872 (80.1)	1925 (87.1)	211 (79.3)	380 (89.4)	**<** **0.001**
Regular follow‐up (*n*, %)	2453 (97.8)	2347 (97.9)	288 (98.6)	455 (99.1)	0.262

Abbreviations: BMI, body mass index; CKD, chronic kidney disease; CVD, cardiovascular disease; DBP, diastolic blood pressure; eGFR, estimated glomerular filtration rate; HDL‐C, high‐density lipoprotein cholesterol; HRQoL, health‐related quality of life; LDL‐C, low‐density lipoprotein cholesterol; PHQ‐9, patient health questionnaire‐9; SBP, systolic blood pressure; SH, severe hypoglycaemia; uACR, urine albumin‐to‐creatinine ratio.

^*^ *p*‐value indicates the differences between the non‐depressed‐low‐HVS, non‐depressed‐high‐HVS, depressed‐low‐HVS and depressed‐high‐HVS groups; *p*‐values less than 0.05 are shown in bold.

### 4.2 Individual Risk of Depression and High HVS on Clinical Outcomes

**FIGURE 2:**
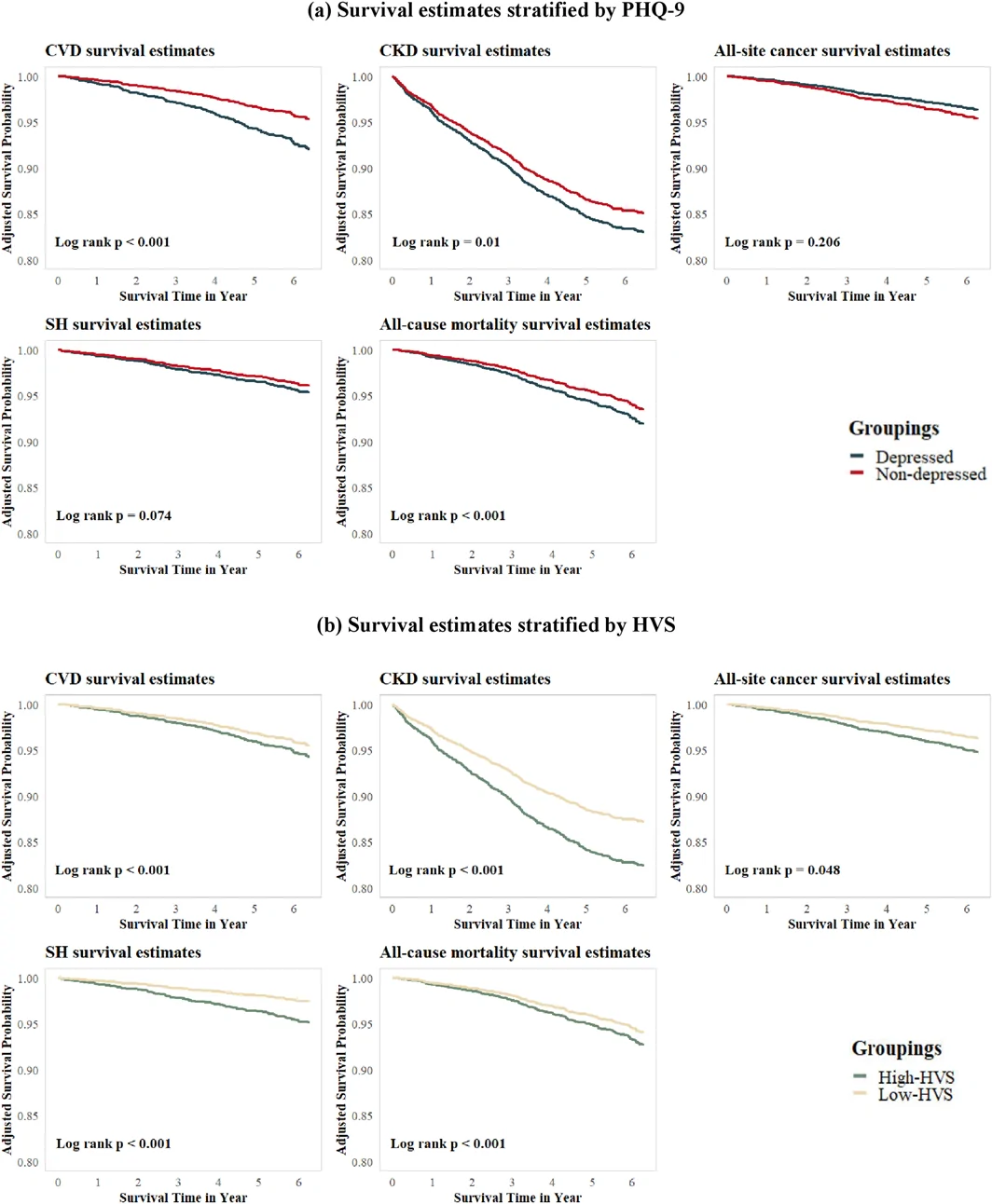
The Kaplan–Meier survival curves of events stratified by (a) depression (PHQ9 ≥ 7) and (b) median value of HVS (≥ 42.9%) on diabetes‐related clinical outcomes. CKD, chronic kidney disease; CVD, cardiovascular disease; HVS, HbA_1c_ variability score; PHQ‐9, patient health questionnaire‐9; SH, severe hypoglycaemia.

In multivariate analyses, depression was associated with an increased risk of CVD and this association remained significant after adjustment for all covariates (aHR: 1.79, 95% CI: 1.15–2.79, *p* = 0.010). The associations of depression with CKD and all‐cause mortality were attenuated and rendered non‐significant after adjusting for self‐management behaviours and HRQoL (Figure 2a). High HVS was independently associated with increased risks of CKD and SH. These associations were attenuated but remained significant after adjusting for self‐management behaviours and HRQoL (aHR for CKD: 1.49, 95% CI: 1.21–1.83, *p* < 0.001; aHR for SH: 1.93, 95% CI: 1.22–3.07, *p* = 0.005). High HVS tended to be associated with increased risk all‐site cancer, albeit short of significance after adjustment of all covariates (Figure 2b).

### 4.3 Joint Associations of Depression and High HVS on Clinical Outcomes

**FIGURE 3:**
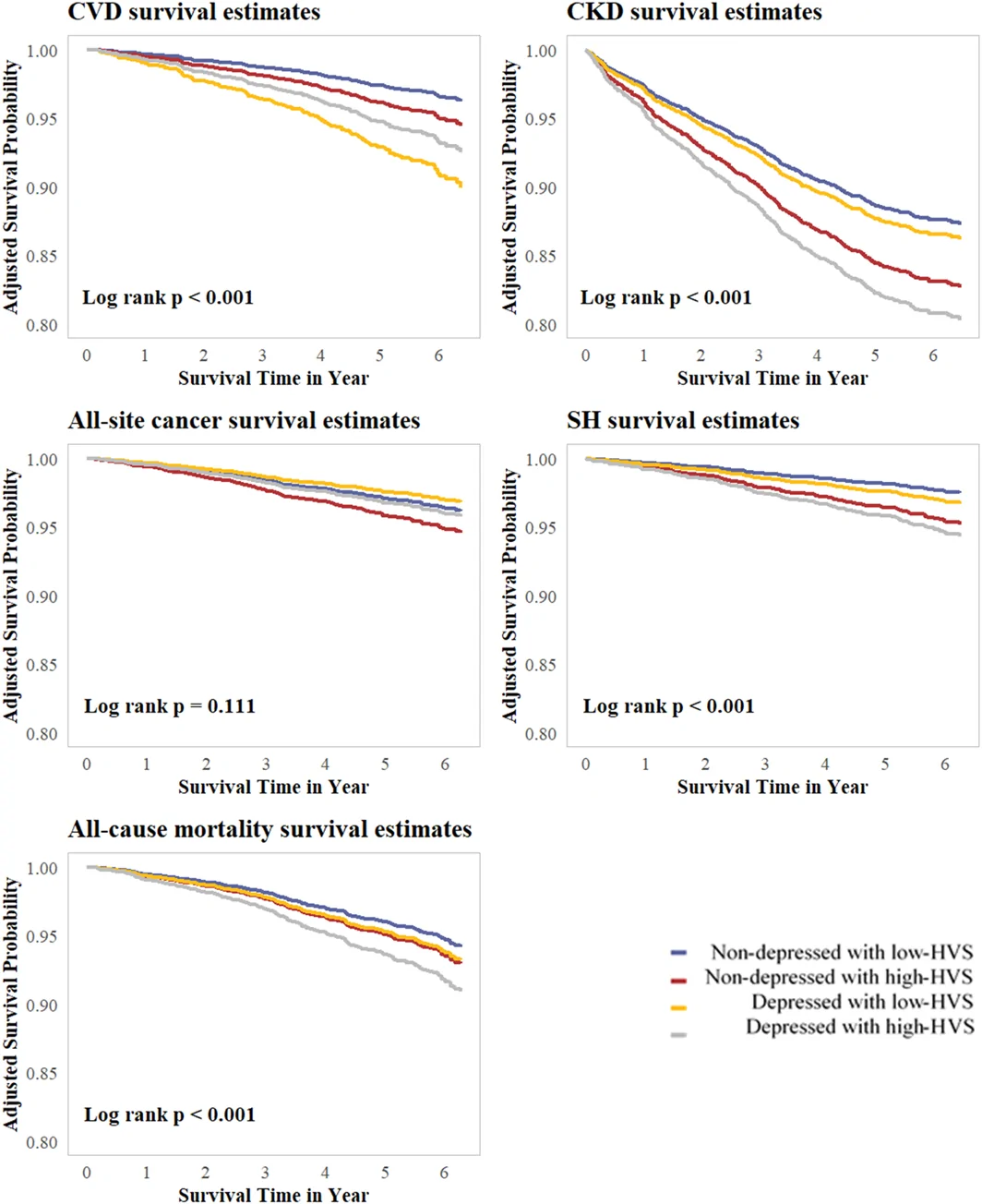
The Kaplan–Meier survival curves of events stratified by depression (PHQ‐9 ≥ 7) with median HVS (≥ 42.9%). CKD, chronic kidney disease; CVD, cardiovascular disease; HVS, HbA_1c_ variability score; SH, severe hypoglycaemia. uACR and eGFR were removed from the model of CKD.

Figure 3 presents the Kaplan–Meier survival curves for clinical outcomes stratified by four groups of depression status and HVS. Compared with the non‐depressed‐low‐HVS group, the depressed‐high‐HVS group had higher risks of CKD, SH and all‐cause mortality. No significant interaction was observed between depression and HVS. After adjustment for self‐management behaviours and HRQoL, the aHR for CKD in the depressed‐high‐HVS group was attenuated from 1.90 (95% CI: 1.41–2.56, *p* < 0.001) to 1.74 (95% CI: 1.25–2.44, *p* = 0.001), and that for all‐cause mortality from 2.00 (95% CI: 1.33–3.01, *p* = 0.001) to 1.72 (95% CI: 1.06–2.78, *p* = 0.028), whereas that with SH increased from 2.23 (95% CI: 1.17–4.24, *p* = 0.015) to aHR of 2.34 (95% CI: 1.16–4.70, *p* = 0.017). For CVD, elevated risks were observed in both depressed‐high‐HVS (aHR: 2.08, 95% CI: 1.08–4.01, *p* = 0.028) and depressed‐low‐HVS groups (aHR: 2.90, 95% CI: 1.49–5.67, *p* = 0.002), which remained significant after multivariable adjustment. No significant associations were found for all‐site cancer.

### 4.4 Sensitivity Analysis

High HbA_1c_‐SD and HbA_1c_‐CV showed patterns similar to HVS with respect to demographic characteristics, cardiometabolic risk profiles and medication use (Supporting Information S1: Tables S5, S6a and S6b). In multivariable analyses, HbA_1c_‐CV yielded results comparable to HVS, with significant risk associations with CKD and SH (Supporting Information S1: Figure S1a). There was a significant association between HbA_1c_‐SD and all‐site cancer but not CKD or SH (Supporting Information S1: Table S7). When HbA_1c_‐SD and HbA_1c_‐CV were used as alternative measures of GV and stratified jointly with depression, findings were broadly consistent with those based on HVS, albeit subtle differences. The non‐depressed‐high‐SD and non‐depressed‐high‐CV groups had the highest risk of all‐site cancer (*p* < 0.05) (Supporting Information S1: Figure S1a,b). No significant difference was observed amongst the four groups when using SD as the stratification variable to predict the risk of SH (Supporting Information S1: Figure S1a). All aHRs for outcomes remained unchanged after adjusting for competing risk of mortality (Supporting Information S1: Table S8).

## 5 Discussion

In this prospective analysis of 5657 Chinese patients with T2D, 13.2% had depressive symptoms (PHQ‐9 ≥ 7), 60% of whom had high HVS. Within the high HVS group, 16.1% had depression, supporting the frequent co‐occurrence of these high‐risk conditions. During the 5‐year follow‐up period (2013–2019), depression was independently associated with CVD, whereas high‐HVS was associated with increased risks of CKD, SH and all‐cause mortality. Concurrent depression and high HVS amplified these risks (except for CVD), which were attenuated by adjusting for self‐management and HRQoL, albeit remaining significant. This study is the first to evaluate the joint associations of depression and glycaemic variability in a well‐characterised prospective Asian cohort with T2D. The persistence of excess risk despite near‐universal healthcare access underscores unmet psychosocial and metabolic needs in T2D.

### 5.1 Depression, Irrespective of HVS, Is Associated With CVD

In this cohort, depression was associated with a two‐fold increased risk of CVD, respectively, with HVS status. In a general Chinese cohort that included both people with and without diabetes, depression using a lower PHQ‐9 cut‐off (> 4, validated primarily in European populations) was associated with 69% increased risk of CVD [24]. Using a higher cut‐off of ≥ 7, in this cohort with T2D, the prevalence of depression (13%) was double that of the earlier general Chinese Cohort (6.46%). Within the same ethnic group, cultural or linguistic attributes may influence screening performance with differences in prevalence of depression [25]. Heterogeneity in covariates including comorbidities may underlie differences in effect sizes of depression with outcomes [26, 27].

Depression is a multifactorial disorder with negative biological and behavioural effects [2, 28]. Depression‐associated obesity and physical inactivity may be accompanied by dysregulation of neurohormonal and immune systems, which can promote cardiometabolic dysfunction and atherosclerosis. In patients with diabetes, depression may lead to suboptimal self‐management including treatment non‐adherence and default of medical follow‐up [29]. In a large meta‐analysis involving two million patients with diabetes, GV was associated with an increased risk of CVD [30]. However, in our detailed analysis with adjustment for confounders including risk factors, comorbidities and treatment, we did not find an association of CVD with GV using all three measures. On the other hand, we found a consistent association between depression and CVD after adjusting for cardiometabolic factors and medications (statins and RASi). In support of our findings, Mendelian Randomisation (MR) analysis suggested that depression is on the causal pathway to CVD, calling for further investigations on the underlying biology and genetic mechanisms [31].

### 5.2 Depression and HVS in Relation to CKD, SH and All‐Cause Mortality

Depression and high HVS represent overlapping yet distinct high‐risk states in patients with T2D. In people with T2D, the risk of depression with mortality increased from 1.67 for mild depression to 2.30 for severe depression [32]. Behavioural factors aside [32, 33], the abnormal milieu associated with depression may cause widespread organ damage with premature death. In our cohort, participants with depression were more likely to be physically inactive with suboptimal treatment adherence and poor HRQoL, despite similar follow‐up intensity. In support of our hypothesis, the highest mortality was found in the depression‐high‐HVS group, which was attenuated after adjusting for self‐management and HRQoL but remained significant. These results suggest that these factors might modulate the impacts of high HVS on outcomes, especially in the presence of depression. In a population‐based cohort of young‐onset diabetes, one‐third of hospital bed‐days were attributable to mental health diagnoses [34, 35]. These young patients also had high glycaemic burden and poor glycaemic control, calling for better understanding of their behavioural health and quality of life [36].

In the aforementioned meta‐analysis, HVS was associated with increased risk of SH, CKD and premature death [30]. We were able to replicate these findings in our cohort. Although depression per se was not associated with these outcomes, the risk was markedly elevated in the presence of high HVS. Poor and fluctuating glycaemic control can increase oxidative stress, endothelial dysfunction, chronic inflammation, platelet activation, impaired angiogenesis and increased fibrosis [37]. Besides, swings in blood glucose level can increase advanced glycation end‐products (AGEs) and activate renin‐angiotensin‐aldosterone system (RAAS) to alter renal haemodynamics [38, 39, 40]. Recurrent hyper‐ and hypoglycaemia disrupt counter‐regulatory responses with increased risk of hypoglycaemic unawareness and SH [41]. Whilst the hazards of CKD in the depression‐high‐HVS group were partially confounded by self‐management and HRQoL, the further increase in hazards of SH and all‐cause mortality events suggests that other factors, such as treatment regimen or therapeutic intensity, may contribute to this excess risk. As per Rothman's multicausality concept [42], complex diseases arise from different component causes, which need to act in concert to cause disease manifestation. Depression alone may be insufficient to substantially increase mortality risk, but in the presence of marked glycaemic instability, vulnerability is amplified. These findings highlight the complex interplay amongst psychological and behavioural health on glycaemic control and clinical outcomes.

### 5.3 Depression and HVS in Relation to All‐Site Cancer

Neither depression nor high HVS was independently associated with all‐site cancer in our primary analyses. Amongst the four groups stratified by depression and HVS, the non‐depressed/high‐HVS group had the highest cancer risk although this was not significant when compared with the non‐depressed/low‐HVS group. However, this association was significant when GV was expressed as HbA_1c‐_SD and HbA_1c_‐CV.

Large fluctuations in blood glucose may promote tumour development and metastasis through oxidative stress, inflammation and metabolic disturbances, suggesting that glycaemic instability beyond average glucose levels may contribute to cancer risk [43, 44]. Current evidence on depression and cancer remains inconclusive [45]. Some studies suggested anti‐depressant use might mitigate cancer risk through anti‐inflammatory actions [46, 47]. Others proposed behavioural and lifestyle factors as mediators of the depression–cancer association [48]. Unlike the consistent causal link between depression and CVD supported by MR analysis using genetic instruments [31], similar analyses examining depression and cancer have yielded inconsistent findings. Taken together, our results suggest that older age, long disease duration and high glycaemic burden with variability may be more important determinants of cancer risk than depression per se [49]. Because of the small sample size, we could not explore the cancer–depression associations for cancer subtypes.

### 5.4 Study Strengths and Limitations

This is the first study which evaluates the joint risk associations of depression and HVS with cardiovascular‐kidney‐cancer events in T2D. The HKDR has provided real‐world data in Chinese patients with diabetes and near‐continuous follow‐up since 1995. Data completeness was high, with only 5% missing data, which is substantially lower than many other registers [50]. The combination of near‐universal healthcare coverage and a territory‐wide electronic medical record system minimises biases due to lack of access to care or data gaps [51]. Since 2013, we systematically collected PROs including HRQoL and PHQ‐9. Together with retrieval of time‐varying laboratory results, we were able to identify patients with high HVS and depression. We evaluated GV using three indices: HVS (primary), HbA_1c_‐SD and HbA_1c_‐CV (sensitivity analyses). Whilst findings were largely consistent, there were subtle differences likely due to different calculation methods [52], calling for cautious interpretation.

Several limitations merit consideration. Depression was identified using PHQ‐9 rather than formal psychiatric diagnosis, which may introduce misclassification. However, most practice guidelines support using simple tools (e.g., PHQ‐9) to identify psychosocial needs in busy clinic settings. Time‐dependent PROs were not examined, and depressive symptoms may vary over time, which could influence risk estimates. All patients were referred to a hospital‐based diabetes centre for structured assessment and management. This may limit generalisability to primary care settings. Excluded patients due to prior events or incomplete data had higher baseline risk profiles, suggesting that our analysis may underestimate the true effect size of the observed risks.

## 6 Conclusion

In this prospective cohort of Chinese patients with T2D, one in eight patients with T2D had depression and more than 50% had fluctuating HbA_1c_. These overlapping but distinct traits were independently and differentially associated with adverse outcomes. Depression was independently associated with CVD, whereas the associations of HVS with SH, CKD and all‐cause mortality were amplified when depression coexisted, albeit attenuated to some extent by self‐management and HRQoL. Our results highlight the complexity, heterogeneity and uncertainties in T2D. Systemic evaluation of biomedical‐psychological‐behavioural risks, particularly in patients with unstable glycaemic control, may identify high‐risk patients for holistic management to improve outcomes.

## Author Contributions

All authors have made substantial contributions to conception and design, revised the article and approved the final version to be published. K.T.C.W. additionally contributed to data analysis and interpretation and wrote the manuscript. J.C.N.C. and J.N.M.L. contributed to the conceptualisation and supervision of this paper and provided critical revision of the manuscript for important intellectual content. E.S.H.L. contributed to data management, curation and cleaning. All other co‐authors have contributed to data collection and discussion. All authors approved the submission of the paper with J.C.N.C. as the guarantor of the data integrity.

## Funding

This project was supported by the RGC Area of Excellence Scheme (AoE/M‐401/24‐R).

## Ethics Statement

The study was approved by the Joint Chinese University of Hong Kong–New Territories East Cluster Clinical Research Ethics Committee (CREC Ref. No. 2007.339).

## Conflicts of Interest

Juliana N.M. Lui has received investigator‐initiated research grant from Roche Diagnostics and Johnson and Johnson unrelated to this study. Juliana C.N. Chan reported receiving grants and/or honoraria for consultancy or giving lectures from Applied Therapeutics, AstraZeneca, Bayer, Boehringer Ingelheim, Celltrion, Eli Lilly, Hua Medicine, Powder Pharmaceuticals, Merck, MSD, Pfizer, Sanofi, Servier, Viatris and Zuellig Pharma. She is the chief executive officer (pro bono) of the Asia Diabetes Foundation that developed the web‐based JADE platform for implementation of data‐driven diabetes care. Alice P.S. Kong has received honorarium for consultancy or giving lectures from Abbott, Astra Zeneca, Bayer, Biomea Fusion Inc, Boehringer Ingelheim, Dexcom, Eli‐Lilly, Kyowa Kirin, Merck Serono, Merck Sharp & Dohme, Nestle, Novo‐Nordisk, Pfizer, Sanofi and Zuellig Pharma. Andrea Luk has received research grants and/or honorarium and/or served as a member of advisory panel for Amgen, AstraZeneca, Bayer, Boehringer Ingelheim, Lee's Pharmaceutical, MSD, Novo Nordisk, Roche, Sanofi, Sugardown Ltd and Takeda, outside the submitted work. Edith W.K. Chow has received travel sponsorships from Boehringer Ingelheim and Novo‐Nordisk, outside the submitted work. The other authors declare no conflict of interest. Ronald C.W. Ma has received research grants and/or honoraria for consultancy or giving lectures from AstraZeneca, Boehringer Ingelheim, Bayer, Kyowa Kirin, Merck, Novo Nordisk, Pfizer, Roche Diagnostics and Tricida Inc. The proceeds have been donated to the Chinese University of Hong Kong, American Diabetes Association and other charitable organizations to support diabetes research and education.

## Supporting information


Supporting Information S1

## Data Availability

Because of legal restrictions, patient‐level data cannot be made publicly available. Aggregated data may be available upon reasonable request with research proposal. Requests to access the dataset should be directed to jchan@cuhk.edu.hk.
