# Supplementary material for: Depression and Long‐Term HbA1c Variability Are Additively Associated With Increased Risk of Cardiovascular–Renal Outcomes, Severe Hypoglycaemia and Mortality in Type‐2 Diabetes
Source: Diabetes Metab Res Rev. 2026 Aug 31;42(6):e70222. doi: 10.1002/dmrr.70222 (PMC13528269; doi:10.1002/dmrr.70222)
Supplement: Supplementary file 1 [file DMRR-42-e70222-s001.docx]

**Depression and Long-term HbA_1c_ Variability are Additively Associated with Increased Risk of Cardiovascular-renal Outcomes, Severe Hypoglycaemia and Mortality in Type-2 Diabetes**

**Supplementary Materials**

**Table of Contents**

**Table S1:** Definitions of study variables	3

**Table S2.** Study variables entered into the stepwise cox regression model	6

**Table S3.** Clinical characteristics between patients included and excluded for analysis in this cohort	7

**Table S4.** Clinical characteristics of included patients, stratified by PHQ9≥7 and median value of HVS (≥42.9%)	9

**Table S5.** Clinical characteristics of patients with type 2 diabetes, stratified by median value of HbA_1c_-SD (≥0.8) and median value of HbA_1c_-CV (≥10.4)	11

**Table S6a.** Clinical characteristics of patients with type 2 diabetes, stratified by PHQ9≥7 and median value of HbA_1c_-CV (≥10.4)	13

**Table S6b.** Clinical characteristics of patients with type 2 diabetes, stratified by PHQ9≥7 and median value of HbA_1c_-SD (≥0.8)	15

**Figure S1a*.*** The Kaplan-Meier survival curve of events stratified by depression (PHQ9≥7) with median HbA_1c_-SD (≥0.8).	17

**Figure S1b*.*** The Kaplan-Meier survival curve of events stratified by depression (PHQ9≥7) with median HbA_1c_-CV (≥10.4).	18

**Table S7.** Cox regression model stratified by median value of HbA_1c_-SD (≥0.8) and HbA_1c_-CV (≥10.4) on diabetes-related clinical outcomes	19

**Table S8.** Cox regression model stratified by PHQ9≥7 with median value of HVS (≥42.9%), median value of HbA_1c_-SD (≥0.8) and HbA_1c_-CV (≥10.4) on diabetes-related clinical outcomes, adjusted for competing risk of death	20

## **Table S1:** Definitions of study variables

| **Study variables** | | | **Categories (*if categorical*)** | **Remarks** |
| --- | --- | --- | --- | --- |
| ***Demographics, socio-economic status and social habits*** | | | | |
|  | Age | |  |  |
|  | Sex | | - Female - Male |  |
|  | Family history of diabetes | | - Yes - No |  |
|  | Follow-up duration | |  |  |
|  | Educational level | | - College or above - Below college |  |
|  | Tobacco use | | - Current smoker / Ex-smoker - Non-smoker |  |
|  | Alcohol use | | - Regular drinker / Occasional / Ex-drinker - Never drank alcohol |  |
|  | Adherence to balanced diet in last 3 months | | - Never / No / Occasional - Yes |  |
|  | Regular physical activity in last 3 months | | - No regular physical activity - Less than 3 times per week - 3-4 times per week - 5 times or more per week | At least 150 minutes per session |
| ***Metabolic risk factors*** | | | | |
|  | Age at diagnosis | |  |  |
|  | Duration of diabetes | |  |  |
|  | HbA_1c_ | |  | Retrieved from first enrolment date to the earliest date of CVD, CHD, CKD, all-site cancer, or death being identified or the censor date of 31st December 2019, whichever came earlier. The HbA_1c_ 2 years priors to the clinical event were excluded from the analysis to avoid reverse causality. |
|  | Time-weighted mean HbA_1c_ | |  | Calculated using Area Under Curve (AUC) |
|  | HVS | |  |  |
|  | HbA_1c_-SD | |  |  |
|  | HbA_1c_-CV | |  |  |
|  | BMI | |  |  |
|  | Waist-hip-ratio | |  |  |
|  | SBP | |  |  |
|  | DBP | |  |  |
|  | HDL-C | |  |  |
|  | LDL-C | |  |  |
|  | Triglyceride | |  |  |
|  | uACR | |  |  |
|  | eGFR | |  |  |
| ***Medications*** | | | | |
|  | Oral glucose lowering drugs | | - Yes - No | Alpha-glucosidase inhibitor (AGIs), dipeptidyl peptidase 4 (DPP-4) inhibitor, meglitinide, metformin, sodium-glucose cotransporter-2 (SGLT2) inhibitor, sulphonylurea, thiazolidinedione |
|  | Insulin | | - Yes - No |  |
|  | Lipid lowering drugs | | - Yes - No | Ezetimibe, cholestyramine, fibrate, statin |
|  | Blood pressure lowering drugs | | - Yes - No | Alpha blocker, beta blocker, calcium channel blocker, hydralazine, methyldopa, nitrate, thiazide, angiotensin-converting enzyme inhibitors (ACEi), angiotensin receptor blockers (ARBs) |
|  | Statin | | - Yes - No |  |
|  | Renin angiotensin system inhibitor | | - Yes - No |  |
| ***Complications and comorbidities*** | | | | |
|  | CVD | | - Yes - No |  |
|  | CKD | | - Yes - No |  |
|  | All-site cancer | | - Yes - No |  |
|  | SH | | - Yes - No |  |
|  | All-cause mortality | | - Yes - No |  |
| ***Patient-reported outcomes*** | | | | |
|  | PHQ-9 | | - PHQ-9 < 7 - PHQ-9 ≥ 7 |  |
|  | EQ-5D-3L | |  |  |
|  |  | Mobility | - I have no problems in walking about - I have some problems in walking about - I am confined to bed |  |
|  |  | Self-care | - I have no problems with self-care - I have some washing or dressing myself - I am unable to wash or dress myself |  |
|  |  | Usual activates | - I have no problems with performing my usual activities - I have some problems with performing my usual activities - I am unable to perform my usual activities |  |
|  |  | Pain/discomfort | - I have no pain or discomfort - I have moderate pain or discomfort - I have extreme pain or discomfort |  |
|  | Self-reported medication adherence | |  | Scoring ranges from 0 to 100 |
|  | Self-manage blood glucose | | - Yes - No |  |
|  | Regular follow-up | | - Yes - No |  |

*Note.* BMI = Body Mass Index; HDL-C = High-density lipoprotein cholesterol; LDL-C = Low-density lipoprotein cholesterol; SBP = Systolic blood pressure; DBP = Diastolic blood pressure; uACR = urine albumin-to-creatinine ratio; eGFR = estimated glomerular filtration rate; HRQoL = health-related quality of life; PHQ-9 = Patient Health Questionnaire-9; HVS = HbA_1c_ variability score; HbA_1c_-SD = HbA_1c_ standard deviation; HbA_1c_-CV = HbA_1c_ coefficient of variation; CVD = Cardiovascular disease; CKD = Chronic kidney disease; SH = Severe hypoglycaemia

# **Table S2.** Study variables entered into the stepwise cox regression model

| **Model** | **Included Study Variables*** |
| --- | --- |
| Model 1 | *Demographics* (age, sex, education level, and duration of diabetes) + *clinical risk factors* (BMI, triglyceride, HDL-C, LDL-C, SBP, DBP, uACR, eGFR, HbA_1c_ at baseline and time-weighted HbA_1c_) |
| Model 2 | *Model 1 + medications* (insulin, oral glucose lowering drugs, BP lowering drugs and lipid lowering drugs) |
| Model 3 | *Model 2 + self-management behaviours* (balanced diet, physical activity, medication adherence, self-manage blood glucose and regular follow-up) |
| Model 4 | *Model 3 + HRQoL* (EQ-5D-3L) |
| Model 5 | *Model 4* + *PHQ-9* (for individual cox regression on HVS and outcomes) **OR**  *Model 4* + *HVS (HbA_1c_-SD / HbA_1c_-CV)* (for individual cox regression on PHQ-9 and outcomes) |

*Note.* BMI = Body Mass Index; HDL-C = High-density lipoprotein cholesterol; LDL-C = Low-density lipoprotein cholesterol; SBP = Systolic blood pressure; DBP = Diastolic blood pressure; uACR = urine albumin-to-creatinine ratio; eGFR = estimated glomerular filtration rate; HRQoL = health-related quality of life; PHQ-9 = Patient Health Questionnaire-9; HVS = HbA_1c_ variability score; HbA_1c_-SD = HbA_1c_ standard deviation; HbA_1c_-CV = HbA_1c_ coefficient of variation

# **Table S3.** Clinical characteristics between patients included and excluded for analysis in this cohort

|  |  |  | **Frequency (%) / Mean±SD / Median (IQR)** | | |  |
| --- | --- | --- | --- | --- | --- | --- |
|  |  |  | **n** | **Excluded subjects (n=1,514)** | **Included subjects (n=5,657)** | **p-value*** |
| **Demographics, socio-economic status and social habits** | | | | | | |
| Age (years) | | | 7,171 | 55.9±13.8 | 58.5±10.6 | **<0.001** |
| Men (n, %) | | | 7,171 | 974 (64.3) | 3322 (58.7) | **<0.001** |
| College or above (n, %) | | | 7,171 | 435 (28.7) | 925 (16.4) | **<0.001** |
| Current smoker (n, %) | | | 7,161 | 201 (13.3) | 623 (11.0) | **0.016** |
| Never drank alcohol (n, %) | | | 7,152 | 801 (53.0) | 3378 (59.9) | **<0.001** |
| Adherent to balanced diet in last 3 months (n, %) | | | 7,147 | 699 (46.3) | 2946 (52.3) | **<0.001** |
| Physical activity 5 times or more per week in last 3 months (n, %) | | | 7,108 | 443 (29.7) | 1969 (35.1) | **<0.001** |
| **Metabolic risk factors** | | |  |  |  |  |
| Age at diagnosis (years) | | | 7,133 | 50.0 (40.0, 58.0) | 48.0 (41.0, 55.0) | **0.004** |
| Duration of diabetes (years) | | | 7,133 | 5.0 (1.0, 13.0) | 9.0 (4.0, 16.0) | **<0.001** |
| Fasting plasma glucose (mmol/L) | | | 7,146 | 7.5±2.8 | 7.6±2.5 | 0.192 |
| HbA_1c_ measurements (times) | | | 6,777 | 3.0 (2.0, 10.3) | 18.0 (10.0, 29.0) | **<0.001** |
| Time-weighted mean HbA_1c_ (%) | | | 6,611 | 7.4±1.3 | 7.4±1.0 | 0.803 |
| HVS (%) | | | 6,051 | 33.3 (0.0, 50.0) | 42.9 (26.7, 56.5) | **<0.001** |
| HbA_1c_-SD (%) | | | 6,217 | 0.6 (0.3, 1.2) | 0.8 (0.5. 1.2) | **<0.001** |
| HbA_1c_-CV (%) | | | 6,217 | 8.2 (4.4, 14.3) | 10.4 (7.2, 15.0) | **<0.001** |
| BMI (kg/m^2^) | | | 7,165 | 25.4±4.2 | 26.5±4.8 | **<0.001** |
| Waist-hip-ratio (women) | | | 2,855 | 0.89±0.08 | 0.92±0.07 | **<0.001** |
| Waist-hip-ratio (men) | | | 4,269 | 0.96±0.07 | 0.97±0.07 | **<0.001** |
| SBP (mmHg) | | | 7,167 | 130.6±19.1 | 133.3±17.9 | **<0.001** |
| DBP (mmHg) | | | 7,162 | 76.1±11.1 | 75.2±10.9 | **0.004** |
| HDL-C (mmol/L) | | | 7,131 | 1.3±0.4 | 1.3±0.4 | **<0.001** |
| LDL-C (mmol/L) | | | 6,961 | 2.4±0.8 | 2.2±0.7 | **<0.001** |
| Triglyceride (mmol/L) | | | 7,156 | 1.2 (0.9, 1.8) | 1.3 (0.9, 1.9) | **0.001** |
| uACR (mg/mmoL) | | | 7,124 | 1.5 (0.6, 8.7) | 1.7 (0.6, 7.8) | 0.188 |
| eGFR (mL/min/1.73m­^2^) | | | 7,166 | 81.6±27.3 | 83.7±23.3 | **0.004** |
| **Medications (n, %)** | | |  |  |  |  |
| Oral glucose lowering drugs | | | 7,171 | 1094 (72.3) | 5012 (88.6) | **<0.001** |
| Insulin | | | 7,171 | 358 (23.6) | 1883 (33.3) | **<0.001** |
| Lipid lowering drugs | | | 7,171 | 807 (53.3) | 3836 (67.8) | **<0.001** |
| Blood pressure lowering drugs | | | 7,171 | 910 (60.1) | 4208 (74.4) | **<0.001** |
| Statin | | | 7,171 | 744 (50.1) | 3576 (64.2) | **<0.001** |
| Renin angiotensin system inhibitor | | | 7,171 | 663 (44.8) | 3206 (57.2) | **<0.001** |
| **Incident of complications and comorbidities (n, %)** | | | | | | |
| Cardiovascular disease | | | 7,171 | 141 (9.3) | 406 (7.2) | **0.006** |
| Chronic kidney disease | | | 7,171 | 543 (35.9) | 1515 (26.8) | **<0.001** |
| All-site cancer | | | 7,171 | 89 (5.9) | 236 (4.2) | **0.006** |
| Severe hypoglycaemia | | | 7,171 | 48 (3.2) | 244 (4.3) | 0.054 |
| All-cause mortality | | | 7,171 | 115 (7.6) | 298 (5.3) | **0.001** |
| **Patient-reported outcomes** | | |  |  |  |  |
| PHQ-9 (0-27) | | | 7,103 | 2.0 (0.0, 4.0) | 2.0 (0.0, 4.0) | 0.455 |
|  | PHQ ≥ 7 (n, %) | | 7,103 | 179 (12.4) | 751 (13.3) | 0.391 |
| EQ-5D-3L (dimension 1-4) (n, %) | | |  |  |  |  |
|  | No problems in walking about | | 7,055 | 1303 (92.3) | 5173 (91.7) | 0.489 |
|  | No problems with self-care | | 7,047 | 1373 (97.4) | 5510 (97.7) | 0.592 |
|  | No problems with performing usual activities | | 7,049 | 1314 (93.4) | 5250 (93.1) | 0.697 |
|  | No pain or discomfort | | 7,041 | 941 (66.9) | 3378 (60.0) | **<0.001** |
| Self-reported medication adherence (0-100) | | | 6,844 | 100.0 (90.0, 100.0) | 100.0 (90.0, 100.0) | 0.346 |
| Self-manage blood glucose (n, %) | | | 6,666 | 1068 (74.8) | 4388 (83.8) | **<0.001** |
| Regular follow-up (n, %) | | | 7,167 | 1396 (92.3) | 5543 (98.0) | **<0.001** |

*Note.* BMI = Body Mass Index; HDL-C = High-density lipoprotein cholesterol; LDL-C = Low-density lipoprotein cholesterol; SBP = Systolic blood pressure; DBP = Diastolic blood pressure; uACR = urine albumin-to-creatinine ratio; eGFR = estimated glomerular filtration rate; HRQoL = health-related quality of life; PHQ-9 = Patient Health Questionnaire-9; HVS = HbA_1c_ variability score; HbA_1c_-SD = HbA_1c_ standard deviation; HbA_1c_-CV = HbA_1c_ coefficient of variation; CVD = Cardiovascular disease; CKD = Chronic kidney disease; SH = Severe hypoglycaemia

## **Table S4.** Clinical characteristics of included patients, stratified by PHQ9≥7 and median value of HVS (≥42.9%)

|  |  |  |  | **Freq (%) / Mean±SD / Median (IQR)** | | | **Freq (%) / Mean±SD / Median (IQR)** | | |
| --- | --- | --- | --- | --- | --- | --- | --- | --- | --- |
|  |  |  | **n** | **Non-depressed (n=4,906)** | **Depressed (n=751)** | **p-value**^†^ | **Low-HVS (n=2,800)** | **High-HVS (n=2,857)** | **p-value**^†^ |
| **Demographics, socio-economic status and social habits** | | |  |  |  |  |  |  |  |
| Age (years) | | | 5,657 | 58.5±10.6 | 58.1±10.7 | 0.334 | 59.2±10.0 | 57.7±11.1 | **<0.001** |
| Men (n, %) | | | 5,657 | 2945 (60.0) | 377 (50.2) | **<0.001** | 1630 (58.2) | 1692 (59.2) | 0.457 |
| College or above (n, %) | | | 5,657 | 837 (17.2) | 88 (11.8) | **<0.001** | 522 (18.8) | 403 (14.2) | **<0.001** |
| Current smoker (n, %) | | | 5,657 | 536 (10.9) | 87 (11.6) | 0.636 | 237 (8.5) | 386 (13.5) | **<0.001** |
| Never drank alcohol (n, %) | | | 5,657 | 2890 (58.9) | 488 (65.0) | **0.003** | 1670 (59.6) | 1708 (59.8) | 0.886 |
| Adherent to balanced diet in last 3 months (n, %) | | | 5,636 | 2561 (52.4) | 385 (51.5) | 0.666 | 1454 (52.1) | 1492 (52.4) | 0.837 |
| Physical activity 5 times or more per week in last 3 months (n, %) | | | 5,614 | 1761 (36.1) | 208 (28.1) | **<0.001** | 1026 (36.8) | 943 (33.4) | **0.009** |
| **Metabolic risk factors** | | |  |  |  |  |  |  |  |
| Age at diagnosis (years) | | | 5,652 | 49.0 (41.0, 56.0) | 47.0 (39.0, 55.0) | **0.001** | 51.0 (44.0, 58.0) | 46.0 (38.0, 52.0) | **<0.001** |
| Duration of diabetes (years) | | | 5,652 | 9.0 (4.0, 15.00) | 10.0 (5.0, 17.0) | **0.001** | 7.0 (3.0, 13.0) | 12.0 (6.0, 18.0) | **<0.001** |
| Fasting plasma glucose (mmol/L) | | | 5,640 | 7.6±2.4 | 7.9±3.3 | **0.001** | 7.0±1.8 | 8.3±3.0 | **<0.001** |
| Time-weighted mean HbA_1c_ (%) | | | 5,657 | 7.4±1.0 | 7.7±1.3 | **<0.001** | 6.9±0.7 | 8.0±1.0 | **<0.001** |
| % of patients with high HVS | | | 5,657 | 2398 (48.9) | 459 (61.1) | **<0.001** | 0 (0.0) | 2857 (100.0) | **<0.001** |
| BMI (kg/m^2^) | | | 5,651 | 26.5±4.7 | 27.0±4.9 | **0.006** | 26.1±4.6 | 27.0±4.9 | **<0.001** |
| Waist-hip-ratio (women) | | | 2,321 | 0.92±0.07 | 0.94±0.08 | **<0.001** | 0.91±0.07 | 0.93±0.07 | **<0.001** |
| Waist-hip-ratio (men) | | | 3,303 | 0.97±0.07 | 0.98±0.07 | **0.001** | 0.96±0.06 | 0.98±0.07 | **<0.001** |
| SBP (mmHg) | | | 5,654 | 133.0±17.7 | 135.0±19.4 | **0.005** | 131.9±17.4 | 134.6±18.4 | **<0.001** |
| DBP (mmHg) | | | 5,649 | 75.1±10.8 | 75.7±11.4 | 0.145 | 75.0±10.7 | 75.3±11.2 | 0.32 |
| HDL-C (mmol/L) | | | 5,632 | 1.3±0.4 | 1.3±0.4 | 0.348 | 1.4±0.4 | 1.3±0.4 | **<0.001** |
| LDL-C (mmol/L) | | | 5,495 | 2.2±0.7 | 2.3±0.8 | 0.502 | 2.2±0.7 | 2.2±0.7 | 0.612 |
| Triglyceride (mmol/L) | | | 5,647 | 1.3 (0.9, 1.9) | 1.4 (1.0, 2.0) | **<0.001** | 1.2 (0.9, 1.8) | 1.3 (1.0, 2.0) | **<0.001** |
| uACR (mg/mmoL) | | | 5,617 | 1.6 (0.6, 7.2) | 2.8 (0.8, 16.6) | **<0.001** | 1.2 (0.5, 4.1) | 2.8 (0.8, 14.3) | **<0.001** |
| eGFR (mL/min/1.73m­^2^) | | | 5,652 | 84.2±22.6 | 80.2±27.6 | **<0.001** | 85.6±19.8 | 81.7±26.2 | **<0.001** |
| **Medications (n, %)** | | |  |  |  |  |  |  |  |
| Oral glucose lowering drugs | | | 5,657 | 4358 (88.8) | 654 (87.1) | 0.18 | 2419 (86.4) | 2593 (90.8) | **<0.001** |
| Insulin | | | 5,657 | 1558 (31.8) | 325 (43.3) | **<0.001** | 451 (16.1) | 1432 (50.1) | **<0.001** |
| Lipid lowering drugs | | | 5,657 | 3314 (67.5) | 522 (69.5) | 0.304 | 1870 (66.8) | 1966 (68.8) | 0.109 |
| Blood pressure lowering drugs | | | 5,657 | 3627 (73.9) | 581 (77.4) | 0.05 | 2003 (71.5) | 2205 (77.2) | **<0.001** |
| Statin | | | 5,571 | 3092 (64.0) | 484 (65.6) | 0.42 | 1760 (64.0) | 1816 (64.4) | 0.738 |
| Renin angiotensin system inhibitor | | | 5,602 | 2768 (57.0) | 438 (59.0) | 0.306 | 1435 (51.8) | 1771 (62.6) | **<0.001** |
| **Incident of complications and comorbidities (n, %)** | | |  |  |  |  |  |  |  |
| CVD | | | 5,657 | 318 (6.5) | 88 (11.7) | **<0.001** | 145 (5.2) | 261 (9.1) | **<0.001** |
| CKD | | | 5,657 | 1261 (25.7) | 254 (33.8) | **<0.001** | 566 (20.2) | 949 (33.2) | **<0.001** |
| All-site cancer | | | 5,657 | 215 (4.4) | 21 (2.8) | 0.054 | 97 (3.5) | 139 (4.9) | **0.01** |
| SH | | | 5,657 | 190 (3.9) | 54 (7.2) | **<0.001** | 53 (1.9) | 191 (6.7) | **<0.001** |
| All-cause mortality | | | 5,657 | 229 (4.7) | 69 (9.2) | **<0.001** | 100 (3.6) | 198 (6.9) | **<0.001** |
| **Patient-reported outcomes** | | |  |  |  |  |  |  |  |
| PHQ-9 (0-27) | | | 5,657 | 1.0 (0.0, 3.0) | 9.0 (7.0, 12.0) | **<0.001** | 1.0 (0.0, 4.0) | 2.0 (0.0, 5.0) | **<0.001** |
|  | PHQ ≥ 7 (n, %) | | 5,657 | 0 (0.0) | 751 (100.0) | **<0.001** | 292 (10.4) | 459 (16.1) | **<0.001** |
| EQ-5D-3L (dimension 1-4) (n, %) | | |  |  |  |  |  |  |  |
|  | No problems in walking about | | 5,657 | 4617 (94.1) | 556 (74.0) | **<0.001** | 2600 (92.9) | 2573 (90.1) | **<0.001** |
|  | No problems with self-care | | 5,657 | 4819 (98.2) | 691 (92.0) | **<0.001** | 2740 (97.9) | 2770 (97.0) | 0.052 |
|  | No problems with performing usual activities | | 5,657 | 4693 (95.7) | 557 (74.2) | **<0.001** | 2632 (94.0) | 2618 (91.6) | **0.001** |
|  | No pain or discomfort | | 5,657 | 3154 (64.3) | 224 (29.8) | **<0.001** | 1695 (60.5) | 1683 (58.9) | 0.251 |
| Self-reported medication adherence (0-100) | | | 5,478 | 100.0 (90.0 100.0) | 98.0 (81.3, 100.0) | **<0.001** | 100.0 (90.0, 100.0) | 99.0 (90.0, 100.0) | **<0.001** |
| Self-manage blood glucose (n, %) | | | 5,238 | 3797 (83.5) | 591 (85.5) | 0.198 | 2083 (80.1) | 2305 (87.4) | **<0.001** |
| Regular follow-up (n, %) | | | 5,657 | 4800 (97.9) | 743 (98.9) | 0.073 | 2741 (97.9) | 2802 (98.1) | 0.694 |

^†^p-value indicates the differences between non-depressed and depressed groups and between low HVS and high HVS group respectively.

*Note.* BMI = Body Mass Index; HDL-C = High-density lipoprotein cholesterol; LDL-C = Low-density lipoprotein cholesterol; SBP = Systolic blood pressure; DBP = Diastolic blood pressure; uACR = urine albumin-to-creatinine ratio; eGFR = estimated glomerular filtration rate; HRQoL = health-related quality of life; PHQ-9 = Patient Health Questionnaire-9; HVS = HbA_1c_ variability score; CVD = Cardiovascular disease; CKD = Chronic kidney disease; SH = Severe hypoglycaemia

## **Table S5.** Clinical characteristics of patients with type 2 diabetes, stratified by median value of HbA_1c_-SD (≥0.8) and median value of HbA_1c_-CV (≥10.4)

|  |  |  |  | **Freq (%) / Mean±SD / Median (IQR)** | | | **Freq (%) / Mean±SD / Median (IQR)** | | |
| --- | --- | --- | --- | --- | --- | --- | --- | --- | --- |
|  |  |  | **n** | **Low-SD**  **(n=2,828)** | **High-SD**  **(n=2,829)** | **p-value**^†^ | **Low-CV**  **(n=2,828)** | **High-CV**  **(n=2,829)** | **p-value**^†^ |
| **Demographics, socio-economic status and social habits** | | | | | | | | | |
| Age (years) | | | 5,657 | 60.3±9.6 | 56.7±11.3 | **<0.001** | 60.3±9.5 | 56.6±11.3 | **<0.001** |
| Men (n, %) | | | 5,657 | 1618 (57.2) | 1704 (60.2) | **0.023** | 1602 (56.6) | 1720 (60.8) | **0.002** |
| College or above (n, %) | | | 5,625 | 499 (17.8) | 426 (15.1) | **0.009** | 491 (17.5) | 434 (15.4) | **0.043** |
| Current smoker (n, %) | | | 5,657 | 239 (8.5) | 384 (13.6) | **<0.001** | 242 (8.6) | 381 (13.5) | **<0.001** |
| Never drank alcohol (n, %) | | | 5,657 | 1692 (59.8) | 1686 (59.6) | 0.957 | 1721 (60.9) | 1657 (58.6) | 0.096 |
| Adherent to balanced diet in last 3 months (n, %) | | | 5,636 | 1430 (50.8) | 1516 (53.8) | **0.025** | 1414 (50.2) | 1532 (54.4) | **0.002** |
| Physical activity 5 times or more per week in last 3 months (n, %) | | | 5,614 | 1049 (37.2) | 920 (32.9) | **0.001** | 1040 (36.9) | 929 (33.2) | **0.004** |
| **Metabolic risk factors** | | |  |  |  |  |  |  |  |
| Age at diagnosis (years) | | | 5,652 | 51.0 (43.0, 57.0) | 46.0 (38.0, 53.0) | **<0.001** | 50.0 (43.0, 57.0) | 46.0 (39.0, 53.0) | **<0.001** |
| Duration of diabetes (years) | | | 5,652 | 8.0 (4.0, 15.0) | 10.0 (4.0, 16.3) | **<0.001** | 9.0 (4.0, 15.0) | 10.0 (4.0, 16.0) | **0.019** |
| Fasting plasma glucose (mmol/L) | | | 5,640 | 7.1±1.8 | 8.2±3.0 | **<0.001** | 7.2±1.9 | 8.1±3.0 | **<0.001** |
| Time-weighted mean HbA_1c_ (%) | | | 5,657 | 7.0±0.7 | 7.9±1.1 | **<0.001** | 7.1±0.9 | 7.8±1.1 | **<0.001** |
| BMI (kg/m^2^) | | | 5,651 | 26.0±4.4 | 27.1±5.1 | **<0.001** | 26.0±4.4 | 27.1±5.1 | **<0.001** |
| Waist-hip-ratio (women) | | | 2,321 | 0.91±0.07 | 0.93±0.07 | **<0.001** | 0.91±0.07 | 0.93±0.07 | **<0.001** |
| Waist-hip-ratio (men) | | | 3,303 | 0.97±0.07 | 0.98±0.07 | **<0.001** | 0.97±0.06 | 0.98±0.07 | **<0.001** |
| SBP (mmHg) | | | 5,654 | 132.5±17.4 | 134.0±18.4 | **0.002** | 132.5±17.3 | 134.0±18.5 | **0.001** |
| DBP (mmHg) | | | 5,649 | 74.8±10.6 | 75.5±11.2 | **0.026** | 74.7±10.6 | 75.6±11.2 | **0.005** |
| HDL-C (mmol/L) | | | 5,632 | 1.3±0.4 | 1.3±0.4 | **<0.001** | 1.3±0.4 | 1.3±0.4 | **<0.001** |
| LDL-C (mmol/L) | | | 5,495 | 2.2±0.7 | 2.3±0.8 | **0.011** | 2.2±0.7 | 2.3±0.8 | 0.075 |
| Triglyceride (mmol/L) | | | 5,647 | 1.3 (0.9, 1.8) | 1.3 (0.9, 2.0) | **<0.001** | 1.3 (0.9, 1.8) | 1.3 (0.9, 2.0) | **<0.001** |
| uACR (mg/mmoL) | | | 5,617 | 1.3 (0.6, 4.6) | 2.6 (0.8, 13.2) | **<0.001** | 1.3 (0.6, 5.0) | 2.5 (0.7, 12.7) | **<0.001** |
| eGFR (mL/min/1.73m­^2^) | | | 5,652 | 84.0±20.2 | 83.3±26.1 | 0.199 | 84.2±20.2 | 83.1±26.1 | 0.071 |
| **Medications (n, %)** | | |  |  |  |  |  |  |  |
| Oral glucose lowering drugs | | | 5,657 | 2466 (87.2) | 2546 (90.0) | **0.001** | 2475 (87.5) | 2537 (89.7) | **0.012** |
| Insulin | | | 5,657 | 520 (18.4) | 1363 (48.2) | **<0.001** | 598 (21.1) | 1285 (45.4) | **<0.001** |
| Lipid lowering drugs | | | 5,657 | 1947 (68.8) | 1889 (66.8) | 0.101 | 1962 (69.4) | 1874 (66.2) | **0.013** |
| Blood pressure lowering drugs | | | 5,657 | 2112 (74.7) | 2096 (74.1) | 0.632 | 2118 (74.9) | 2090 (73.9) | 0.398 |
| Statin | | | 5,571 | 1834 (65.9) | 1742 (62.5) | **0.008** | 1849 (66.4) | 1727 (61.9) | **0.001** |
| Renin angiotensin system inhibitor | | | 5,602 | 1531 (54.6) | 1675 (59.9) | **<0.001** | 1544 (55.1) | 1662 (59.3) | **0.002** |
| **Incident of complications and comorbidities (n, %)** | | | | | | | | | |
| CVD | | | 5,657 | 164 (5.8) | 242 (8.6) | **<0.001** | 166 (5.9) | 240 (8.5) | **<0.001** |
| CKD | | | 5,657 | 655 (23.2) | 860 (30.4) | **<0.001** | 658 (23.3) | 857 (30.3) | **<0.001** |
| All-site cancer | | | 5,657 | 107 (3.8) | 129 (4.6) | 0.163 | 111 (3.9) | 125 (4.4) | 0.389 |
| SH | | | 5,657 | 67 (2.4) | 177 (6.3) | **<0.001** | 69 (2.4) | 175 (6.2) | **<0.001** |
| All-cause mortality | | | 5,657 | 114 (4.0) | 184 (6.5) | **<0.001** | 114 (4.0) | 184 (6.5) | **<0.001** |
| **Patient-reported outcomes** | | |  |  |  |  |  |  |  |
| PHQ-9 (0-27) | | | 5,657 | 1.0 (0.0, 4.0) | 2.0 (0.0, 5.0) | **<0.001** | 1.0 (0.0, 4.0) | 2.0 (0.0, 5.0) | **<0.001** |
|  | PHQ ≥ 7 (n, %) | | 5,657 | 296 (10.5) | 455 (16.1) | **<0.001** | 299 (10.6) | 452 (16.0) | **<0.001** |
| EQ-5D-3L (dimension 1-4) (n, %) | | |  |  |  |  |  |  |  |
|  | No problems in walking about | | 5,657 | 2611 (92.3) | 2562 (90.6) | **0.023** | 2617 (92.5) | 2556 (90.3) | **0.006** |
|  | No problems with self-care | | 5,657 | 2769 (97.9) | 2741 (96.9) | **0.025** | 2771 (98.0) | 2739 (96.8) | **0.015** |
|  | No problems with performing usual activities | | 5,657 | 2659 (94.0) | 2591 (91.6) | **0.001** | 2663 (94.2) | 2587 (91.4) | **<0.001** |
|  | No pain or discomfort | | 5,657 | 1682 (59.5) | 1696 (60.0) | 0.748 | 1685 (59.6) | 1693 (59.8) | 0.875 |
| Self-reported medication adherence (0-100) | | | 5,478 | 100.0 (90.0, 100.0) | 99.0 (90.0, 100.0) | **<0.001** | 100.0 (90.0, 100.0) | 99.0 (90.0, 100.0) | **<0.001** |
| Self-manage blood glucose (n, %) | | | 5,238 | 2084 (79.2) | 2304 (88.4) | **<0.001** | 2104 (79.8) | 2284 (87.7) | **<0.001** |
| Regular follow-up (n, %) | | | 5,657 | 2778 (98.2) | 2765 (97.8) | 0.293 | 2778 (98.2) | 2765 (97.8) | 0.293 |

^†^p-value indicates the differences between low SD and high SD and between low CV and high CV group respectively.

*Note.* BMI = Body Mass Index; HDL-C = High-density lipoprotein cholesterol; LDL-C = Low-density lipoprotein cholesterol; SBP = Systolic blood pressure; DBP = Diastolic blood pressure; uACR = urine albumin-to-creatinine ratio; eGFR = estimated glomerular filtration rate; HRQoL = health-related quality of life; PHQ-9 = Patient Health Questionnaire-9; HVS = HbA_1c_ variability score; HbA_1c_-SD = HbA_1c_ standard deviation; HbA_1c_-CV = HbA_1c_ coefficient of variation; CVD = Cardiovascular disease; CKD = Chronic kidney disease; SH = Severe hypoglycaemia

## **Table S6a.** Clinical characteristics of patients with type 2 diabetes, stratified by PHQ9≥7 and median value of HbA_1c_-CV (≥10.4)

|  |  |  | **Freq (%) / Mean±SD / Median (IQR)** | | | |  |
| --- | --- | --- | --- | --- | --- | --- | --- |
|  |  |  | **Non-depressed & Low-CV**  **(n=2,529)** | **Non-depressed & High-CV**  **(n=2,377)** | **Depressed & Low-CV**  **(n=299)** | **Depressed & High-CV**  **(n=452)** | **p-value**^†^ |
| **Demographics, socio-economic status and social habits** | | | | | | | |
| Age (years) | | | 60.3±9.5 | 56.6±11.4 | 60.3±9.8 | 56.7±11.0 | **<0.001** |
| Men (n, %) | | | 1469 (58.1) | 1476 (62.1) | 133 (44.5) | 244 (54.0) | **<0.001** |
| College or above (n, %) | | | 455 (18.0) | 382 (16.1) | 36 (12.0) | 52 (11.5) | **0.001** |
| Current smoker (n, %) | | | 228 (9.0) | 308 (13.0) | 14 (4.7) | 73 (16.2) | **<0.001** |
| Never alcohol drinker (n, %) | | | 1512 (59.9) | 1378 (58.2) | 209 (69.9) | 279 (61.7) | **0.001** |
| Adherent to balanced diet in last 3 months (n, %) | | | 1271 (50.4) | 1290 (54.5) | 143 (48.0) | 242 (53.8) | **0.014** |
| Physical activity 5 times or more per week in last 3 months (n, %) | | | 954 (37.8) | 807 (34.3) | 86 (29.2) | 122 (27.5) | **<0.001** |
| **Metabolic risk factors** | | |  |  |  |  |  |
| Age at diagnosis (years) | | | 50.0 (43.0, 57.0) | 47.0 (39.0, 53.0) | 50.0 (41.5, 56.0) | 46.0 (37.8, 52.3) | **<0.001** |
| Duration of diabetes (years) | | | 8.0 (4.0, 15.0) | 10.0 (4.0, 16.0) | 9.0 (5.0, 16.0) | 11.0 (5.0, 17.0) | **0.001** |
| Fasting plasma glucose (mmol/L) | | | 7.2±1.9 | 8.1±2.8 | 7.2±2.3 | 8.4±3.7 | **<0.001** |
| Time-weighted mean HbA_1c_ (%) | | | 7.1±0.8 | 7.8±1.1 | 7.2±1.0 | 8.0±1.3 | **<0.001** |
| BMI (kg/m^2^) | | | 26.0±4.3 | 27.0±5.1 | 26.2±4.7 | 27.5±5.1 | **<0.001** |
| Waist-hip-ratio (women) | | | 0.91±0.07 | 0.93±0.07 | 0.92±0.08 | 0.95±0.08 | **<0.001** |
| Waist-hip-ratio (men) | | | 0.96±0.06 | 0.98±0.07 | 0.98±0.07 | 0.99±0.07 | **<0.001** |
| SBP (mmHg) | | | 132.4±17.2 | 133.6±18.1 | 133.3±18.3 | 136.1±20.1 | **<0.001** |
| DBP (mmHg) | | | 74.8±10.5 | 75.4±11.2 | 74.7±11.3 | 76.3±11.4 | **0.015** |
| HDL-C (mmol/L) | | | 1.3±0.4 | 1.3±0.4 | 1.4±0.4 | 1.2±0.4 | **<0.001** |
| LDL-C (mmol/L) | | | 2.2±0.7 | 2.3±0.7 | 2.3±0.7 | 2.2±0.8 | **0.043** |
| Triglyceride (mmol/L) | | | 1.2 (0.9, 1.8) | 1.3 (0.9, 1.9) | 1.4 (1.0, 1.9) | 1.4 (1.0, 2.1) | **<0.001** |
| uACR (mg/mmoL) | | | 1.2 (0.6, 4.5) | 2.3 (0.7, 11.8) | 1.7 (0.7, 8.9) | 3.6 (0.9, 22.5) | **<0.001** |
| eGFR (mL/min/1.73m­^2^) | | | 84.5±19.7 | 83.8±25.3 | 81.6±23.9 | 79.3±29.8 | **<0.001** |
| **Medications (n, %)** | | |  |  |  |  |  |
| Oral glucose lowering drugs | | | 2215 (87.6) | 2143 (90.2) | 260 (87.0) | 394 (87.2) | **0.019** |
| Insulin | | | 523 (20.7) | 1035 (43.5) | 75 (25.1) | 250 (55.3) | **<0.001** |
| Lipid lowering drugs | | | 1748 (69.1) | 1566 (65.9) | 214 (71.6) | 308 (68.1) | **0.046** |
| Blood pressure lowering drugs | | | 1885 (74.5) | 1742 (73.3) | 233 (77.9) | 348 (77.0) | 0.163 |
| Statin | | | 1648 (66.2) | 1444 (61.7) | 201 (68.8) | 283 (63.5) | **0.004** |
| Renin angiotensin system inhibitor | | | 1379 (55.1) | 1389 (59.0) | 165 (55.6) | 273 (61.3) | **0.011** |
| **Incident of complications and comorbidities (n, %)** | | |  |  |  |  |  |
| CVD | | | 140 (5.5) | 178 (7.5) | 26 (8.7) | 62 (13.7) | **<0.001** |
| CKD | | | 564 (22.3) | 697 (29.3) | 94 (31.4) | 160 (35.4) | **<0.001** |
| All-site cancer | | | 99 (3.9) | 116 (4.9) | 12 (4.0) | 9 (2.0) | **0.032** |
| SH | | | 60 (2.4) | 130 (5.5) | 9 (3.0) | 45 (10.0) | **<0.001** |
| All-cause mortality | | | 93 (3.7) | 136 (5.7) | 21 (7.0) | 48 (10.6) | **<0.001** |
| **Patient-reported outcomes** | | |  |  |  |  |  |
| PHQ-9 (0-27) | | | 1.0 (0.0, 3.0) | 1.0 (0.0, 3.0) | 9.0 (7.0, 11.0) | 9.00 (8.0, 12.0) | **<0.001** |
| EQ-5D-3L (dimension 1-4) (n, %) | | |  |  |  |  |  |
|  | No problems in walking about | | 2401 (95.1) | 2216 (93.5) | 216 (72.7) | 340 (75.6) | **<0.001** |
|  | No problems with self-care | | 2492 (98.8) | 2327 (98.3) | 279 (93.6) | 412 (91.6) | **<0.001** |
|  | No problems with performing usual activities | | 2442 (96.7) | 2251 (95.0) | 221 (74.2) | 336 (74.7) | **<0.001** |
|  | No pain or discomfort | | 1601 (63.6) | 1553 (65.6) | 84 (28.3) | 140 (31.1) | **<0.001** |
| Self-reported medication adherence (0-100) | | | 100.0 (90.0, 100.0) | 100.0 (90.0, 100.0) | 99.0 (90.0, 100.0) | 95.0 (80.0, 100.0) | **<0.001** |
| Self-manage blood glucose (n, %) | | | 1880 (79.7) | 1917 (87.6) | 224 (80.9) | 367 (88.6) | **<0.001** |
| Regular follow-up (n, %) | | | 2481 (98.1) | 2319 (97.6) | 297 (99.3) | 446 (98.7) | 0.14 |

^†^p-value indicates the differences between non-depressed-low-CV, non-depressed-high-CV, depressed-low-CV and depressed-high-CV groups.

*Note.* BMI = Body Mass Index; HDL-C = High-density lipoprotein cholesterol; LDL-C = Low-density lipoprotein cholesterol; SBP = Systolic blood pressure; DBP = Diastolic blood pressure; uACR = urine albumin-to-creatinine ratio; eGFR = estimated glomerular filtration rate; HRQoL = health-related quality of life; PHQ-9 = Patient Health Questionnaire-9; HVS = HbA_1c_ variability score; HbA_1c_-SD = HbA_1c_ standard deviation; HbA_1c_-CV = HbA_1c_ coefficient of variation; CVD = Cardiovascular disease; CKD = Chronic kidney disease; SH = Severe hypoglycaemia

## **Table S6b.** Clinical characteristics of patients with type 2 diabetes, stratified by PHQ9≥7 and median value of HbA_1c_-SD (≥0.8)

|  |  |  | **Freq (%) / Mean±SD / Median (IQR)** | | | |  |
| --- | --- | --- | --- | --- | --- | --- | --- |
|  |  |  | **Non-depressed & Low-SD**  **(n=2,532)** | **Non-depressed & High-SD**  **(n=2,374)** | **Depressed & Low-SD**  **(n=296)** | **Depressed & High-SD**  **(n=455)** | **p-value**^†^ |
| **Demographics, socio-economic status and social habits** | | | | | | | |
| Age (years) | | | 60.3±9.6 | 56.7±11.3 | 60.3±9.9 | 56.7±10.9 | **<0.001** |
| Men (n, %) | | | 1488 (58.8) | 1457 (61.4) | 130 (43.9) | 247 (54.3) | **<0.001** |
| College or above (n, %) | | | 464 (18.3) | 373 (15.7) | 35 (11.8) | 53 (11.6) | **<0.001** |
| Current smoker (n, %) | | | 228 (9.0) | 308 (13.0) | 11 (3.7) | 76 (16.7) | **<0.001** |
| Never alcohol drinker (n, %) | | | 1488 (58.9) | 1402 (59.3) | 204 (68.9) | 284 (62.4) | **0.006** |
| Adherent to balanced diet in last 3 months (n, %) | | | 1284 (50.9) | 1277 (54.0) | 146 (49.5) | 239 (52.8) | 0.134 |
| Physical activity 5 times or more per week in last 3 months (n, %) | | | 961 (38.1) | 800 (34.0) | 88 (30.0) | 120 (26.9) | **<0.001** |
| **Metabolic risk factors** | | |  |  |  |  |  |
| Age at diagnosis (years) | | | 51.0 (43.0, 57.0) | 46.0 (39.0, 53.0) | 50.0 (42.0, 56.3) | 45.00 (37.0, 52.0) | **<0.001** |
| Duration of diabetes (years) | | | 8.0 (4.0, 15.0) | 10.0 (4.0, 16.0) | 9.0 (4.0, 15.3) | 11.0 (5.0, 18.0) | **<0.001** |
| Fasting plasma glucose (mmol/L) | | | 7.1±1.7 | 8.2±2.8 | 7.1±2.1 | 8.5±3.8 | **<0.001** |
| Time-weighted mean HbA_1c_ (%) | | | 7.0±0.7 | 7.9±1.1 | 7.0±0.7 | 8.2±1.3 | **<0.001** |
| BMI (kg/m^2^) | | | 25.9±4.3 | 27.0±5.1 | 26.3±4.7 | 27.4±5.0 | **<0.001** |
| Waist-hip-ratio (women) | | | 0.91±0.07 | 0.93±0.07 | 0.93±0.08 | 0.95±0.08 | **<0.001** |
| Waist-hip-ratio (men) | | | 0.96±0.06 | 0.98±0.07 | 0.98±0.07 | 0.99±0.07 | **<0.001** |
| SBP (mmHg) | | | 132.3±17.3 | 133.7±18.0 | 133.9±17.9 | 135.7±20.3 | **0.001** |
| DBP (mmHg) | | | 74.8±10.6 | 75.4±11.1 | 75.3±11.3 | 75.9±11.5 | 0.085 |
| HDL-C (mmol/L) | | | 1.3±0.4 | 1.3±0.4 | 1.4±0.4 | 1.3±0.4 | **<0.001** |
| LDL-C (mmol/L) | | | 2.2±0.7 | 2.3±0.8 | 2.3±0.7 | 2.3±0.8 | **0.019** |
| Triglyceride (mmol/L) | | | 1.20 (0.90, 1.80) | 1.30 (0.90, 1.90) | 1.40 (1.00, 2.00) | 1.40 (1.00, 2.00) | **<0.001** |
| uACR (mg/mmoL) | | | 1.20 (0.51, 4.30) | 2.40 (0.70, 12.05) | 1.70 (0.70, 8.07) | 3.60 (0.90, 23.95) | **<0.001** |
| eGFR (mL/min/1.73m­^2^) | | | 84.3±19.7 | 84.0±25.2 | 81.6±23.7 | 79.3±29.8 | **<0.001** |
| **Medications (n, %)** | | |  |  |  |  |  |
| Oral glucose lowering drugs | | | 2209 (87.2) | 2149 (90.5) | 257 (86.8) | 397 (87.3) | **0.002** |
| Insulin | | | 458 (18.1) | 1100 (46.3) | 62 (20.9) | 263 (57.8) | **<0.001** |
| Lipid lowering drugs | | | 1737 (68.6) | 1577 (66.4) | 210 (70.9) | 312 (68.6) | 0.235 |
| Blood pressure lowering drugs | | | 1881 (74.3) | 1746 (73.5) | 231 (78.0) | 350 (76.9) | 0.212 |
| Statin | | | 1637 (65.6) | 1455 (62.2) | 197 (68.2) | 287 (63.9) | **0.041** |
| Renin angiotensin system inhibitor | | | 1367 (54.4) | 1401 (59.6) | 164 (55.8) | 274 (61.2) | **0.001** |
| **Incident of complications and comorbidities (n, %)** | | |  |  |  |  |  |
| CVD | | | 137 (5.4) | 181 (7.6) | 27 (9.1) | 61 (13.4) | **<0.001** |
| CKD | | | 565 (22.3) | 696 (29.3) | 90 (30.4) | 164 (36.0) | **<0.001** |
| All-site cancer | | | 95 (3.8) | 120 (5.1) | 12 (4.1) | 9 (2.0) | **0.011** |
| SH | | | 57 (2.3) | 133 (5.6) | 10 (3.4) | 44 (9.7) | **<0.001** |
| All-cause mortality | | | 94 (3.7) | 135 (5.7) | 20 (6.8) | 49 (10.8) | **<0.001** |
| **Patient-reported outcomes** | | |  |  |  |  |  |
| PHQ-9 (0-27) | | | 1.0 (0.0, 3.0) | 1.0 (0.0, 3.0) | 9.0 (7.0, 11.0) | 9.0 (8.0, 12.0) | **<0.001** |
| EQ-5D-3L (dimension 1-4) (n, %) | | |  |  |  |  |  |
|  | No problems in walking about | | 2397 (94.8) | 2220 (93.8) | 214 (72.8) | 342 (75.5) | **<0.001** |
|  | No problems with self-care | | 2494 (98.8) | 2325 (98.3) | 275 (93.2) | 416 (91.8) | **<0.001** |
|  | No problems with performing usual activities | | 2438 (96.5) | 2255 (95.3) | 221 (74.9) | 336 (74.2) | **<0.001** |
|  | No pain or discomfort | | 1598 (63.4) | 1556 (65.8) | 84 (28.6) | 140 (30.9) | **<0.001** |
| Self-reported medication adherence (0-100) | | | 100.0 (90.0, 100.0) | 100.0 (90.0, 100.0) | 99.0 (90.0, 100.0) | 95.0 (80.0, 100.0) | **<0.001** |
| Self-manage blood glucose (n, %) | | | 1868 (79.3) | 1929 (88.1) | 216 (78.8) | 375 (89.9) | **<0.001** |
| Regular follow-up (n, %) | | | 2484 (98.1) | 2316 (97.6) | 294 (99.3) | 449 (98.7) | 0.14 |

^†^p-value indicates the differences between non-depressed-low-SD, non-depressed-high-SD, depressed-low-SD and depressed-high-SD groups.

*Note.* BMI = Body Mass Index; HDL-C = High-density lipoprotein cholesterol; LDL-C = Low-density lipoprotein cholesterol; SBP = Systolic blood pressure; DBP = Diastolic blood pressure; uACR = urine albumin-to-creatinine ratio; eGFR = estimated glomerular filtration rate; HRQoL = health-related quality of life; PHQ-9 = Patient Health Questionnaire-9; HbA_1c_-SD = HbA_1c_ standard deviation; CVD = Cardiovascular disease; CKD = Chronic kidney disease; SH = Severe hypoglycaemia

## ***Figure S1a.*** The Kaplan-Meier survival curve of events stratified by depression (PHQ9≥7) with median HbA_1c_-SD (≥0.8).

*Note.* HbA_1c_-SD = HbA_1c_ standard deviation; CVD = Cardiovascular disease; CKD = Chronic kidney disease; SH = Severe hypoglycaemia. uACR and eGFR were removed from the model of CKD.

# ***Figure S1b.*** The Kaplan-Meier survival curve of events stratified by depression (PHQ9≥7) with median HbA_1c_-CV (≥10.4).

*Note.* HbA_1c_-CV = HbA_1c_ coefficient of variation; CVD = Cardiovascular disease; CKD = Chronic kidney disease; SH = Severe hypoglycaemia. uACR and eGFR were removed from the model of CKD.

## **Table S7.** Cox regression model stratified by median value of HbA_1c_-SD (≥0.8) and HbA_1c_-CV (≥10.4) on diabetes-related clinical outcomes

|  |  | **Event/**  **Total**^‡^ | **HbA_1c_-SD**  **HR (95% CI)** | **p-value**^†^ | **Event/**  **Total**^‡^ | **HbA_1c_-CV**  **HR (95% CI)** | **p-value**^†^ |
| --- | --- | --- | --- | --- | --- | --- | --- |
| **CVD** | | 106/2244 |  |  | 102/2252 |  |  |
|  | Model 1 |  | 1.19 (0.81, 1.74) | 0.374 |  | 1.06 (0.74, 1.52) | 0.752 |
|  | Model 2 |  | 1.19 (0.81, 1.75) | 0.377 |  | 1.06 (0.74, 1.52) | 0.751 |
|  | Model 3 |  | 1.21 (0.80, 1.82) | 0.368 |  | 1.08 (0.73, 1.58) | 0.713 |
|  | Model 4 |  | 1.24 (0.82, 1.88) | 0.305 |  | 1.10 (0.74, 1.62) | 0.641 |
|  | Model 5 |  | 1.23 (0.81, 1.86) | 0.336 |  | 1.08 (0.73, 1.59) | 0.699 |
| **CKD**^§^ | | 338/2307 |  |  | 332/2304 |  |  |
|  | Model 1 |  | 1.21 (1.00, 1.48) | 0.050 |  | 1.29 (1.07, 1.54) | **0.006** |
|  | Model 2 |  | 1.21 (1.00, 1.47) | 0.054 |  | 1.28 (1.07, 1.54) | **0.006** |
|  | Model 3 |  | 1.22 (0.99, 1.49) | 0.060 |  | 1.28 (1.06, 1.55) | **0.010** |
|  | Model 4 |  | 1.24 (1.01, 1.53) | 0.038 |  | 1.30 (1.07, 1.57) | **0.007** |
|  | Model 5 |  | 1.24 (1.01, 1.52) | 0.040 |  | 1.30 (1.07, 1.57) | **0.008** |
| **All-site cancer** | | 104/2638 |  |  | 100/2638 |  |  |
|  | Model 1 |  | 1.79 (1.26, 2.55) | **0.001** |  | 1.55 (1.11, 2.16) | **0.009** |
|  | Model 2 |  | 1.76 (1.23, 2.51) | **0.002** |  | 1.53 (1.09, 2.13) | **0.013** |
|  | Model 3 |  | 1.55 (1.06, 2.27) | **0.023** |  | 1.37 (0.96, 1.95) | 0.084 |
|  | Model 4 |  | 1.53 (1.04, 2.24) | **0.029** |  | 1.35 (0.95, 1.93) | 0.096 |
|  | Model 5 |  | 1.53 (1.04, 2.24) | **0.029** |  | 1.36 (0.95, 1.94) | 0.094 |
| **SH** | | 103/2346 |  |  | 102/2354 |  |  |
|  | Model 1 |  | 1.58 (1.05, 2.38) | **0.029** |  | 1.73 (1.18, 2.52) | **0.005** |
|  | Model 2 |  | 1.53 (1.02, 2.30) | **0.042** |  | 1.70 (1.16, 2.48) | **0.006** |
|  | Model 3 |  | 1.39 (0.91, 2.14) | 0.130 |  | 1.56 (1.06, 2.32) | **0.025** |
|  | Model 4 |  | 1.39 (0.91, 2.14) | 0.130 |  | 1.56 (1.05, 2.31) | **0.027** |
|  | Model 5 |  | 1.39 (0.91, 2.14) | 0.131 |  | 1.56 (1.05, 2.31) | **0.027** |
| **All-cause mortality** | | 184/2829 |  |  | 184/2829 |  |  |
|  | Model 1 |  | 1.27 (0.95, 1.70) | 0.111 |  | 1.34 (1.02, 1.77) | **0.039** |
|  | Model 2 |  | 1.24 (0.92, 1.67) | 0.151 |  | 1.31 (0.99, 1.74) | 0.057 |
|  | Model 3 |  | 1.26 (0.92, 1.73) | 0.150 |  | 1.35 (1.00, 1.82) | 0.050 |
|  | Model 4 |  | 1.27 (0.92, 1.75) | 0.142 |  | 1.34 (0.99, 1.81) | 0.056 |
|  | Model 5 |  | 1.28 (0.93, 1.76) | 0.134 |  | 1.35 (1.00, 1.82) | 0.052 |

*Note.* HbA_1c_-SD = HbA_1c_ standard deviation; HbA_1c_-CV = HbA_1c_ coefficient of variation; CVD = Cardiovascular disease; CKD = Chronic kidney disease; SH = Severe hypoglycaemia. Model 1 = Adjusted for demographics (age, gender, educational level, duration of diabetes, BMI, HDL-C, LDL-C, SBP, DBP, TG, uACR, eGFR, HbA1c baseline, time-weighted HbA1c); Model 2 = Adjusted for Model 1 + medications (use of insulin, oral glucose lowering drugs, blood pressure lowering drugs, lipid lowering drugs); Model 3 = Adjusted for Model 2 + self-care behaviours (balance diet, physical activities, medication adherence, self-manage blood glucose, regular follow-up); Model 4 = Adjusted for Model 3 + EQ-5D-3L mobility, self-care, usual activities, pain/discomfort; Model 5 = Adjusted for Model 4 + PHQ9 (categorical).

^‡^Event/Total = No. of events among depressed group and high HVS group. ^§^uACR and eGFR were removed from the model of CKD. ^†^p-value indicates the differences between non-depressed and depressed groups and between low HVS and high HVS group respectively. Low HbA_1c_-SD (<0.8) and HbA_1c_-CV (<10.4) groups as referent respectively.

## **Table S8.** Cox regression model stratified by PHQ9≥7 with median value of HVS (≥42.9%), median value of HbA_1c_-SD (≥0.8) and HbA_1c_-CV (≥10.4) on diabetes-related clinical outcomes, adjusted for competing risk of death

|  |  |  | **Event/**  **Total**^‡^ | **Model 1** | | **Model 2** | | **Model 3** | | **Model 4** | |
| --- | --- | --- | --- | --- | --- | --- | --- | --- | --- | --- | --- |
|  |  |  |  | **HR (95%CI)** | **p-value**^†^ | **HR (95%CI)** | **p-value**^†^ | **HR (95%CI)** | **p-value**^†^ | **HR (95%CI)** | **p-value**^†^ |
| **CVD** | | |  |  |  |  |  |  |  |  |  |
| HVS | | |  |  |  |  |  |  |  |  |  |
|  | Non-depressed & Low-HVS | | 48/1980 | (interaction) | 0.092 | (interaction) | 0.089 | (interaction) | 0.067 | (interaction) | 0.100 |
|  | Non-depressed & High-HVS | | 86/1892 | 1.42 (0.94, 2.14) | 0.093 | 1.42 (0.94, 2.15) | 0.093 | 1.52 (0.97, 2.37) | 0.065 | 1.51 (0.96, 2.36) | 0.073 |
|  | Depressed & Low-HVS | | 15/238 | 2.76 (1.52, 5.02) | **0.001** | 2.78 (1.53, 5.04) | **<0.001** | 2.86 (1.50, 5.45) | **0.002** | 2.91 (1.47, 5.75) | **0.002** |
|  | Depressed & High-HVS | | 27/326 | 2.01 (1.08, 3.73) | **0.027** | 2.01 (1.08, 3.74) | **0.028** | 2.00 (1.02, 3.90) | **0.043** | 2.17 (1.09, 4.32) | **0.028** |
| HbA_1c_-SD | | |  |  |  |  |  |  |  |  |  |
|  | Non-depressed & Low-SD | | 56/1963 | (interaction) | 0.450 | (interaction) | 0.450 | (interaction) | 0.170 | (interaction) | 0.170 |
|  | Non-depressed & High-SD | | 78/1909 | 1.27 (0.86, 1.87) | 0.240 | 1.26 (0.85, 1.87) | 0.240 | 1.35 (0.88, 2.06) | 0.170 | 1.37 (0.89, 2.10) | 0.150 |
|  | Depressed & Low-SD | | 14/229 | 2.17 (1.16, 4.06) | **0.015** | 2.18 (1.17, 4.06) | **0.014** | 2.44 (1.27, 4.67) | **0.007** | 2.65 (1.35, 5.19) | **0.005** |
|  | Depressed & High-SD | | 28/335 | 2.02 (1.10, 3.71) | **0.024** | 2.02 (1.10, 3.71) | **0.024** | 1.87 (0.97, 3.64) | 0.063 | 2.02 (1.03, 3.97) | **0.042** |
| HbA_1c_-CV | | |  |  |  |  |  |  |  |  |  |
|  | Non-depressed & Low-CV | | 59/1953 | (interaction) | 0.320 | (interaction) | 0.320 | (interaction) | 0.240 | (interaction) | 0.200 |
|  | Non-depressed & High-CV | | 75/1919 | 1.14 (0.77, 1.68) | 0.510 | 1.14 (0.77, 1.67) | 0.520 | 1.17 (0.78, 1.76) | 0.450 | 1.19 (0.79, 1.80) | 0.400 |
|  | Depressed & Low-CV | | 15/231 | 2.26 (1.26, 4.06) | **0.006** | 2.26 (1.26, 4.06) | **0.006** | 2.31 (1.23, 4.34) | **0.009** | 2.52 (1.31, 4.87) | **0.006** |
|  | Depressed & High-CV | | 27/333 | 1.72 (0.94, 3.16) | 0.078 | 1.72 (0.94, 3.16) | 0.078 | 1.64 (0.86, 3.10) | 0.130 | 1.77 (0.92, 3.38) | 0.087 |
| **CKD**^§^ | | |  |  |  |  |  |  |  |  |  |
| HVS | | |  |  |  |  |  |  |  |  |  |
|  | Non-depressed & Low-HVS | | 234/2239 | (interaction) | 0.660 | (interaction) | 0.500 | (interaction) | 0.650 | (interaction) | 0.780 |
|  | Non-depressed & High-HVS | | 313/1953 | 1.39 (1.12, 1.71) | **0.002** | 1.38 (1.11, 1.70) | **0.00**3 | 1.45 (1.16, 1.80) | **0.001** | 1.48 (1.19, 1.85) | **0.001** |
|  | Depressed & Low-HVS | | 28/257 | 1.14 (0.74, 1.76) | 0.540 | 1.14 (0.74, 1.75) | 0.550 | 1.15 (0.72, 1.81) | 0.560 | 1.08 (0.68, 1.72) | 0.730 |
|  | Depressed & High-HVS | | 70/338 | 1.79 (1.27, 2.51) | **0.001** | 1.88 (1.37, 2.57) | **<0.001** | 1.88 (1.35, 2.63) | **<0.001** | 1.74 (1.21, 2.49) | **0.003** |
| HbA_1c_-SD | | |  |  |  |  |  |  |  |  |  |
|  | Non-depressed & Low-SD | | 264/2231 | (interaction) | 0.260 | (interaction) | 0.390 | (interaction) | 0.520 | (interaction) | 0.620 |
|  | Non-depressed & High-SD | | 283/1961 | 1.26 (1.01, 1.57) | **0.042** | 1.26 (1.01, 1.57) | **0.045** | 1.25 (1.00, 1.58) | 0.053 | 1.29 (1.03, 1.63) | **0.030** |
|  | Depressed & Low-SD | | 43/249 | 1.47 (1.02, 2.12) | **0.037** | 1.46 (1.01, 2.10) | **0.043** | 1.39 (0.94, 2.04) | 0.099 | 1.25 (0.83, 1.86) | 0.280 |
|  | Depressed & High-SD | | 55/346 | 1.39 (0.97, 1.99) | 0.072 | 1.48 (1.07, 2.06) | **0.018** | 1.47 (1.04, 2.09) | **0.029** | 1.42 (0.98, 2.05) | 0.064 |
| HbA_1c_-CV | | |  |  |  |  |  |  |  |  |  |
|  | Non-depressed & Low-CV | | 268/2233 | (interaction) | 0.110 | (interaction) | 0.110 | (interaction) | 0.180 | (interaction) | 0.240 |
|  | Non-depressed & High-CV | | 279/1959 | 1.35 (1.10, 1.66) | **0.004** | 1.35 (1.10, 1.66) | **0.004** | 1.34 (1.09, 1.66) | **0.006** | 1.37 (1.11, 1.69) | **0.003** |
|  | Depressed & Low-CV | | 45/250 | 1.55 (1.09, 2.21) | **0.015** | 1.58 (1.11, 2.25) | **0.011** | 1.51 (1.04, 2.19) | **0.031** | 1.36 (0.92, 2.01) | 0.120 |
|  | Depressed & High-CV | | 53/345 | 1.40 (0.98, 1.99) | 0.066 | 1.46 (1.06, 2.03) | **0.022** | 1.44 (1.02, 2.04) | **0.039** | 1.39 (0.96, 2.00) | 0.084 |
| **All-site cancer** | | |  |  |  |  |  |  |  |  |  |
| HVS | | |  |  |  |  |  |  |  |  |  |
|  | Non-depressed & Low-HVS | | 75/2362 | (interaction) | 0.780 | (interaction) | 0.750 | (interaction) | 0.890 | (interaction) | 0.910 |
|  | Non-depressed & High-HVS | | 95/2233 | 1.46 (1.03, 2.08) | **0.035** | 1.46 (1.02, 2.07) | **0.037** | 1.47 (1.01, 2.15) | **0.043** | 1.46 (1.00, 2.12) | **0.049** |
|  | Depressed & Low-HVS | | 6/263 | 0.74 (0.32, 1.75) | 0.500 | 0.75 (0.32, 1.76) | 0.510 | 0.73 (0.29, 1.89) | 0.520 | 0.81 (0.31, 2.11) | 0.670 |
|  | Depressed & High-HVS | | 12/419 | 0.93 (0.45, 1.95) | 0.850 | 0.92 (0.44, 1.92) | 0.820 | 1.00 (0.46, 2.18) | 1.000 | 1.11 (0.50, 2.50) | 0.800 |
| HbA_1c_-SD | | |  |  |  |  |  |  |  |  |  |
|  | Non-depressed & Low-SD | | 74/2378 | (interaction) | **0.034** | (interaction) | **0.035** | (interaction) | 0.071 | (interaction) | 0.067 |
|  | Non-depressed & High-SD | | 96/2217 | 1.98 (1.36, 2.90) | **<0.001** | 1.95 (1.33, 2.86) | **0.001** | 1.68 (1.11, 2.55) | **0.014** | 1.67 (1.10, 2.54) | **0.017** |
|  | Depressed & Low-SD | | 10/267 | 1.22 (0.61, 2.46) | 0.570 | 1.22 (0.61, 2.46) | 0.580 | 1.19 (0.56, 2.51) | 0.650 | 1.36 (0.63, 2.95) | 0.440 |
|  | Depressed & High-SD | | 8/421 | 0.79 (0.33, 1.87) | 0.590 | 0.77 (0.33, 1.82) | 0.560 | 0.71 (0.28, 1.80) | 0.470 | 0.78 (0.30, 2.02) | 0.620 |
| HbA_1c_-CV | | |  |  |  |  |  |  |  |  |  |
|  | Non-depressed & Low-CV | | 78/2374 | (interaction) | 0.062 | (interaction) | 0.062 | (interaction) | 0.100 | (interaction) | 0.097 |
|  | Non-depressed & High-CV | | 92/2221 | 1.69 (1.18, 2.42) | **0.004** | 1.66 (1.16, 2.39) | **0.006** | 1.46 (0.99, 2.16) | 0.054 | 1.46 (0.98, 2.16) | 0.060 |
|  | Depressed & Low-CV | | 10/271 | 1.12 (0.56, 2.27) | 0.750 | 1.12 (0.56, 2.27) | 0.750 | 1.11 (0.52, 2.33) | 0.790 | 1.27 (0.59, 2.77) | 0.540 |
|  | Depressed & High-CV | | 8/417 | 0.71 (0.30, 1.64) | 0.420 | 0.69 (0.30, 1.59) | 0.390 | 0.64 (0.26, 1.59) | 0.330 | 0.71 (0.28, 1.78) | 0.470 |
| **Severe hypoglycaemia** | | |  |  |  |  |  |  |  |  |  |
| HVS | | |  |  |  |  |  |  |  |  |  |
|  | Non-depressed & Low-HVS | | 32/2348 | (interaction) | 0.810 | (interaction) | 0.800 | (interaction) | 0.890 | (interaction) | 0.890 |
|  | Non-depressed & High-HVS | | 96/2070 | 2.22 (1.36, 3.62) | **0.001** | 2.13 (1.30, 3.49) | **0.003** | 1.97 (1.16, 3.33) | **0.011** | 1.98 (1.17, 3.36) | **0.011** |
|  | Depressed & Low-HVS | | 4/275 | 1.14 (0.40, 3.26) | 0.810 | 1.18 (0.41, 3.36) | 0.760 | 1.20 (0.42, 3.47) | 0.730 | 1.31 (0.46, 3.76) | 0.610 |
|  | Depressed & High-HVS | | 22/362 | 2.20 (1.11, 4.36) | **0.024** | 2.15 (1.09, 4.25) | **0.028** | 2.18 (1.06, 4.51) | **0.034** | 2.40 (1.09, 5.26) | **0.029** |
| HbA_1c_-SD | | |  |  |  |  |  |  |  |  |  |
|  | Non-depressed & Low-SD | | 45/2429 | (interaction) | 0.690 | (interaction) | 0.790 | (interaction) | 0.930 | (interaction) | 0.930 |
|  | Non-depressed & High-SD | | 83/1989 | 1.65 (1.06, 2.56) | **0.025** | 1.58 (1.01, 2.45) | **0.043** | 1.42 (0.89, 2.28) | 0.150 | 1.42 (0.89, 2.29) | 0.140 |
|  | Depressed & Low-SD | | 6/280 | 1.15 (0.48, 2.76) | 0.750 | 1.14 (0.48, 2.73) | 0.770 | 1.15 (0.47, 2.80) | 0.760 | 1.25 (0.51, 3.06) | 0.630 |
|  | Depressed & High-SD | | 20/357 | 1.55 (0.80, 3.01) | 0.200 | 1.56 (0.81, 2.99) | 0.180 | 1.56 (0.77, 3.14) | 0.210 | 1.69 (0.80, 3.57) | 0.170 |
| HbA_1c_-CV | | |  |  |  |  |  |  |  |  |  |
|  | Non-depressed & Low-CV | | 47/2420 | (interaction) | 0.550 | (interaction) | 0.500 | (interaction) | 0.430 | (interaction) | 0.430 |
|  | Non-depressed & High-CV | | 81/1998 | 1.67 (1.12, 2.50) | **0.011** | 1.63 (1.09, 2.43) | **0.017** | 1.48 (0.97, 2.25) | 0.071 | 1.48 (0.96, 2.26) | 0.073 |
|  | Depressed & Low-CV | | 5/281 | 0.78 (0.30, 2.01) | 0.610 | 0.78 (0.31, 2.01) | 0.610 | 0.81 (0.31, 2.10) | 0.660 | 0.88 (0.34, 2.27) | 0.800 |
|  | Depressed & High-CV | | 21/356 | 1.82 (0.98, 3.38) | 0.059 | 1.85 (1.01, 3.40) | **0.046** | 1.87 (0.98, 3.55) | 0.057 | 2.03 (1.00, 4.10) | **0.049** |

*Note.* PHQ-9 = Patient Health Questionnaire-9; HVS = HbA_1c_ variability score; HbA_1c_-SD = HbA_1c_ standard deviation; HbA_1c_-CV = HbA_1c_ coefficient of variation; CVD = Cardiovascular disease; CKD = Chronic kidney disease; SH = Severe hypoglycaemia. Model 1 = Adjusted for demographics (age, gender, educational level, duration of diabetes, BMI, HDL-C, LDL-C, SBP, DBP, TG, uACR, eGFR, HbA_1c_ baseline, time-weighted HbA_1c_); Model 2 = Adjusted for Model 1 + medications (use of insulin, oral glucose lowering drugs, blood pressure lowering drugs, lipid lowering drugs); Model 3 = Adjusted for Model 2 + self-care behaviours (balance diet, physical activities, medication adherence, self-manage blood glucose, regular follow-up); Model 4 = Adjusted for Model 3 + EQ-5D-3L mobility, self-care, usual activities, pain/discomfort.

^‡^Event/Total = No. of events among depressed group and high HVS group. ^§^uACR and eGFR were removed from the model of CKD. ^†^p-value indicates the differences between non-depressed and depressed groups and between low HVS and high HVS group respectively.
